# Subcellular-Resolution Molecular Pathology by Laser Ablation–Rapid Evaporative Ionization Mass Spectrometry

**DOI:** 10.1021/acs.analchem.5c02013

**Published:** 2025-08-06

**Authors:** Dániel Simon, Gabriel Stefan Horkovics-Kováts, Yuchen Xiang, Ronan A. Battle, Yu Wang, Julia Abda, Dimitris Papanastasiou, Stefania Maneta Stavrakaki, Hui-Yu Ho, Haixing Wang, Richard Schäffer, Tamás Karancsi, Anna Mroz, István Pap, Laurine Lagache, Júlia Balog, Isabelle Fournier, Robert T. Murray, Josephine Bunch, Zoltan Takáts

**Affiliations:** † Department of Metabolism, Digestion and Reproduction, 4615 Imperial College London, Exhibition Road, London SW7 2AZ, United Kingdom; ‡ Rosalind Franklin Institute, Fermi Avenue, Didcot OX11 0QS, United Kingdom; § Institut für Funktionelle Genomik, University Regensburg, 9 Am Biopark, Regensburg 93053, Germany; ∥ Hevesy György PhD School of Chemistry, ELTE Eötvös Loránd University 1/A Pázmány Péter sétány, Budapest 1117, Hungary; ⊥ Waters Research Center, 7 Zahony utca, Budapest 1031, Hungary; # Department of Physics, Imperial College London, Exhibition Road, London SW7 2AZ, United Kingdom; g Fasmatech, TESPA Lefkippos, NCSR Demokritos, Athens 15310, Greece; h Department of General Surgery, Linkou Chang Gung Memorial Hospital, No. 5, Fuxing St, Guishan District, Taoyuan City 333, Taiwan; i PRISM Inserm U1192, University of Lille, 42 Rue Paul Duez, Lille 59000, France; j 9917 National Physical Laboratory, Hampton Road, London TW11 0LW, United Kingdom

## Abstract

This work demonstrates the combination of ambient laser ablation (LA) with in-source surface-induced declustering, originally developed for rapid evaporative ionization mass spectrometry (REIMS). This combination, termed laser ablation REIMS (LA-REIMS), provides sensitivity, spatial resolution, and chemical coverage comparable to matrix-assisted laser desoprtion ionization (MALDI) but without the requirement for matrix deposition. The atmospheric pressure interface setup was subjected to detailed characterization with regard to geometric and thermal parameters augmented by in-silico flow modeling. The resulting platform was tested using aerosol formed by the infrared laser ablation of tissues. Three different laser systems were successfully employed for ambient mass spectrometric imaging: a carbon dioxide laser (λ = 10.6 μm, τ_L_ = ∼100 μs), an optical parametric oscillator (OPO; λ = 2.94 μm, τ_L_ = 8 ns), and an optical parametric amplifier (OPA; λ = 3.0 μm, τ_L_ = ∼30 ps). Single-cell imaging was achieved using the high-resolving capabilities of the OPA systems, and metabolites and lipids ranging from amino acids through carbohydrates and nuclear bases to complex glycolipids were successfully detected. The technique was also tested as a platform for MS-guided surgery, raising the possibility of using a single technique for generating histological and in vivo data.

## Graphical abstract

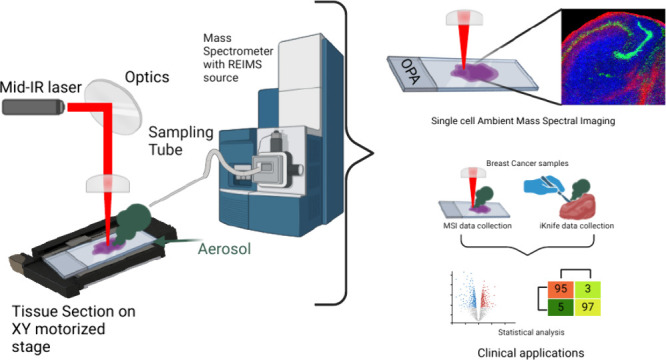

## Introduction

Laser desorption ionization (LDI) mass spectrometry, introduced in the late 1960s,^1,2^ was one of the first laser-based techniques for biomolecular analysis. In the 1970s, LDI was adapted for imaging, initially termed laser microprobe mass analysis (LAMMA).^3,4^ While the rationale of this method for tissue analysis is straightforward, LDI/LAMMA has never gained true popularity as an organic mass spectrometry (MS) method due to a handful of inherent problems stemming from the fundamentals of the underlying biological matter–laser interactions. These challenges are 3-fold. {1} Thermally induced condensation reactions turn biological samples into a three-dimensional, highly cross-linked polymer structure, eventually leading to carbonization and efficiently trapping analyte molecules. {2} The formation of large amounts of neutral molecules in the gas phase relative to the formation of ions. {3} Aerosolization of samples leading to particles containing up to 10^8^–10^9^ molecules.^5–7^ Together, these three effects act against ionization efficiency, making the approach insensitive enough for most biological applications. The first major breakthrough to solve these problems was the development of matrix-assisted LDI (MALDI), where soluble analytes are extracted into a crystalline layer of small organic molecules termed the “matrix”.^8^ The basic requirements for a matrix include high absorbance at the wavelength of the laser and the formation of only gaseous degradation products on laser irradiation.^9^ The MALDI sample preparation process transfers soluble analytes into an environment close to ideal for laser desorption, giving several orders of magnitude improvement on the ionization efficiency of small molecules (metabolites, lipids), while also allowing the ionization of macromolecular species like intact proteins. MALDI also provided a simple means for mass spectrometry imaging (MSI), an area that was predominantly limited to analyzing inorganic compounds by techniques such as secondary ionization mass spectrometry (SIMS). MALDI as an ionization method predominantly tackles problem {1} (with regard to the biological matter–laser interactions described above). It still produces a high amount of neutrals and clusters while introducing a set of new challenges, mainly centered around the deposition of the matrix layer^10^ As a result, all matrix deposition procedures try to establish a compromise between spatial resolution and sensitivity.^11^ Following the development of MALDI, several different approaches have been tested for tackling problem {2} (formation of gas-phase neutrals) in either the presence or absence of the matrix. These methods, collectively termed “postdesorption ionization”, or just “postionization” methods, ranged from photoionization to discharge ionization approaches used in conjunction with LDI. One of the most sophisticated methods, MALDI-2, revolutionized MALDI applications in the course of the last five years by further improving the sensitivity of the technique by 2 to 3 orders of magnitude.^12^ In MALDI-2, the desorption plume is orthogonally irradiated with a high-fluence UV laser to facilitate the photoionization of the matrix followed by chemical ionization-like charge transfer to the analyte molecules. We also demonstrated recently that low-temperature plasma postionization results in a similar sensitivity improvement even in the absence of the matrix.^13^ However, none of these approaches provided a reasonable solution for problem {3}, the formation of large molecular clusters resistant to postionization techniques or the heating of certain parts of the ion path, e.g., the atmospheric interface for AP-MALDI where the cluster formation is particularly profound due to the limited expansion of the desorption plume under high-pressure conditions. The problem of cluster formation is not limited to LDI; practically all methods developed for the ionization of condensed phase samples suffer from this phenomenon, including electrospray, the single most widely used ionization method.^14,15^ One approach to mitigate these phenomena is the orthogonal coupling of the charged droplet stream originating from electrospray with aerosol from the laser desorption process. Electrospray laser desorption techniques (ELDI) have been successfully utilized for analyzing biological material including proteins and small molecules.^16^ Further developments of this concept include laser ablation electrospray ionization (LAESI) which utilizes mid-IR lasers and endogenous water in tissues to perform ablation besides the postdesorption ionization through ESI.^17^ The technique has successfully been utilized for ambient ionization and for single-cell mass spectrometry imaging at 40 μm spatial resolution.^18^ Matrix-assisted laser desorption electrospray ionization (MALDESI) utilizes endogenous or exogenous matrixes and UV or IR lasers to ionize samples.^19^ Generally a thin layer of ice is applied to the tissue surface and mid-IR lasers are utilized for the desorption process, and the method was successfully utilized for high-throughput screening, quantitative analysis,^20^ and tissue imaging applications.^21,22^ The RASTIR technique was also developed to enable long-distance (∼30 cm) ambient sampling and subsequent ionization.^23^ While these techniques provide answers to the cluster formation and ion liberation challenges, their applicability is limited for in vivo applications, for example, during a surgical intervention.

The idea of utilizing surface-induced dissociation to tackle this problem originated from the electrospray droplet impact technique, an approach developed for enhancing the ionization efficiency of electrospray ionization.^24^ Clusters accelerated by the adiabatic expansion following the first gas conductance limit in an atmospheric interface can be impacted against a solid target to induce their dissociation in a straightforward manner. Advantages of the approach include the short time scale compared to slow heating methods and the independence of cluster size compared to gas collision methods. The method works in practice and fulfills the expectations of an efficient gas-phase droplet declustering/ionization method.

Developing a technology equally capable of histological imaging with a view of coregistering the resulting data with classical histopathology and collecting in situ, in vivo data could enable surgeons to operate with histological precision. Rapid evaporative ionization mass spectrometry (REIMS) has been successfully utilized for mass spectrometry-guided surgery applications, ambient food analysis, and the identification and/or biochemical characterization of microorganisms.^25–28^ The original REIMS setup also unitizes an impact declustering-based ionization method, as discussed in our earlier reports. While the benefits of declustering via the heated collision surface were described previously,^29^ the exact aerosol kinetics and the optimal interface geometry were not investigated in detail and to the best of our knowledge there were no prior works on the topic. Atmospheric pressure laser desorption ionization methods, despite their poor sensitivity, have gained momentum following our initial publication on the usage of a number of different laser systems for direct tissue analysis.^30^ The subsequently developed SpiderMass system utilizing resonant IR laser at 3 μm^31^ as well as the CO_2_ laser system^32^ were successfully used for bulk tissue analysis with a clear perspective for direct surgical application, especially in the case of surgical lasers. These systems have also been utilized for imaging, albeit usually with crude resolution, due to the lack of dedicated focal optics.^33^ Further noteworthy development was the use of picosecond infrared lasers for the same purpose resulting in clear surgical advantages, including minimal thermal spread and ultrafine dissections.^34,35^ The utilization of mid-IR ultrafast lasers for mass spectrometry has been also reported recently.^36–39^ The laser ablation process producing the primary aerosol is dependent on the laser parameters including the wavelength, pulse width, and fluence. Several groups published the successful application of different resonant infrared lasers for tissue ablation—mass spectrometry sampling, with some of these lasers being commercially available for medical usage. However, the mechanisms of the resonant ablation and the effects of this on the subsequent ionization remain poorly understood.^40–44^

The current work was aimed at developing a system that is robust and sufficiently sensitive to support mass spectrometry-guided surgical interventions as well as molecular histopathology via MSI. We believe that the proposed technology represents a promising solution for the integration of MSI-based histology and MS-guided surgery. In order to achieve this goal, a three-phase project was conducted. First the atmospheric interface conditions were evaluated and optimized to maximize sensitivity. Second, we coupled various mid-IR laser sources to REIMS to investigate the laser ablation–REIMS postionization method and establish the imaging workflow. Finally, we conducted a proof-of-concept translational study to demonstrate an MSI to an in vivo classification workflow. In detail, we investigated the mechanisms behind surface-induced declustering (SID) present in the experimental atmospheric setup. The numerical models yielded an optimal geometrical configuration for the collision surface-based setup. The sensitivity increase enabled by the source optimization enabled the high-resolution imaging work. These results were used for coupling resonant infrared laser desorption to the postionization method, and we achieved single-cell resolution using a novel picosecond infrared source, enabling us to perform molecular pathology imaging using a sample-preparation free ambient ionization workflow. We also demonstrated the feasibility of our holistic “from molecular pathology to in vivo surgery” diagnostic platform using breast cancer samples, where molecular diagnostics models built from validated MSI data sets were successfully used to classify breast cancer tissues in a setup analogous to in vivo surgical environments.

## Methods and Materials

### Experimental Design

A Waters Xevo G2-XS QToF mass spectrometer (Waters, Wilmslow, U.K.) was used for all of the experiments. The instrument was equipped with a modified REIMS source described by Balog et al.^29^ 100 μL/min MS grade 2 propanol (Merck, Gillingham, UK) was injected in front of the MS inlet capillary to achieve matrix-assisted REIMS as described by Jones et al.^45^ A 1.5 m long, 1.6 mm I.D. PTFE tube (Merck, Gillingham, U.K.) was used to aspirate the aerosol from the sampling position. For the iKnife diathermy experiments, a ForceTriad (Medtronic, Watford, U.K.) electrosurgical generator was used in monopolar mode. The electrosurgical unit was coupled with a modified electrosurgical handpiece (Waters Research Center, Budapest, Hungary), with an output power of 20 W. Pork liver tissues were obtained from commercial suppliers, while mouse brain tissues were obtained as part of a CRUK program. Ethical approval was gained from the South East London Research Ethics Committee Reference 11/LO/0686, the East of England - Cambridge East Research Ethics Committee Reference 14/EE/0024, and the project was registered under the Imperial College Tissue Bank. Data were obtained only from patients who had consented to the use of tissue for research.

### Laser Parameter Characterization and Imaging

A commercially available Opolette HE2731 optical parametric oscillator (Opotek, Carlsbad, USA) and a FELS25A Intelliguide CO_2_ laser (Omniguide, Cambridge, MA, USA) were used during the experiments. The picosecond infrared laser source described by Battle et al.^46^ was used for higher-resolution imaging experiments. Pulses were generated at a repetition rate of 8.3 MHz with broadband spectra at 2950 ± 100 nm. After focusing with an LA5315-E CaF2 lens (Thorlabs), a pulse energy of 12 nJ was delivered to the sample, corresponding to an average power of 100 mW at the sample, which was measured using a PM10 broadband thermopile sensor (Coherent). Two LTS150 (Thorlabs) motorized stages were used in an XY configuration, while an additional MFA-CC (Newport) motorized stage was used to provide fine adjustment along the optical axis. The imaging workflow was otherwise the same as described for the tunable OPO laser source. Optomechanical components were obtained from Thorlabs for the optical cage system construction (Thorlabs, Newton, NJ, USA). LA7733-E4, LA7477-E4 ZnSe, and LA5315-E CaF_2_ lenses were used for the OPO source, and only LA7733-E3 ZnSe was used for the CO_2_ laser due to the lack of an appropriate antireflection coating. The information about the different lenses used can be found in Table S3. To test the effect of wavelength on the tissue desorption efficiency, 12 μm fresh frozen pork liver slides were sampled using the tunable OPO laser between 2700 and 3100 nm, and the laser fluence was normed to 5 J/cm^2^. The laser energy was measured using an EnergyMax-RS J-10MB-HE energy sensor (Coherent, Saxonburg, PA, USA) after the last focal lens on the defocused beam (around 10 mm from the focal spot). The fluences were calculated by measuring the area of the ablated spot sizes and the used energy for ablation using optical microscopy. The imaging experiments were done using a modified two-dimensional stage setup (Prosolia), and the XY motorized stage was removed from the front of the mass spectrometer and mounted on an optics bench. For imaging data processing and visualization of the ion heatmap images, HDImaging (version 1.4, Waters) software was used.

### Statistical Analysis

Data processing for modeling was performed using Abstract Model Builder (AMX, version 1.1967.0, Waters). This software was used to define the spectra collected for data analysis. Mass drift correction was performed against the leucine enkephalin lock mass compound (negative mode *m*/*z* = 554.2615), and mass binning was done to 0.1 Da. For univariate analysis, an in-house Python-based data processing pipeline was used. Principal component analysis (PCA) was also performed to evaluate spectral differences using AMX software. Principal component analysis (PCA) and PCA-linear discriminant analysis (PCA-LDA) models were built, and the PCA-LDA model was used for tissue classification. Spectral comparison for the translational study was performed with multivariate statistical approaches. Both diathermy and laser data were plotted on the same PCA-LDA model, which was cross-validated using a leave-20%-out cross-validation method due to the limited number of breast cancer imaging data. Figures were prepared using Biorender.com.

### Annotations

Metabolite and lipid assignments of the ions detected with LA-REIMS were conducted using in-house-developed and online databases (LIPID MAPS and HMDB), based on accurate mass, isotopic composition, and biological reference. Liquid chromatography-tandem mass spectrometry (LC–MS/MS) was performed by the National Phenome Centre (NPC) to confirm the assignment of a subset of metabolites and lipids. Mouse brain tissue was extracted in 1:5 (v/v) water/propan-2-ol (Optima). Homogenization of the sample was first performed using a Percelis bead beater. The homogenate was then left at 4 °C for 2 h for protein precipitation and subsequently was centrifuged at 10,000 rpm for 5 min. The supernatant was then collected and used for the analysis. For the LC–MS/MS analysis, the NPC’s validated reversed-phase lipidomic assay was used as described by Lewis et al.^47^ Profiling and data-dependent acquisition (DDA) spectra were acquired, and the identification of the ions was performed based on the retention time and the fragmentation pattern.

## Results and Discussion

An experimental platform was developed based on the REIMS ionization method, with the platform and experimental workflow shown in Figure 1. Since the optimization of geometrical parameters is extremely time-consuming due to their high interdependency and the necessity of a venting–pumping cycle following any modifications, we have performed direct Monte Carlo simulations (DMCS) to map the parameter space. Our primary questions were whether there is an optimal overall geometry, including the shape and position of the collision surface. Further questions included the temperature profile of the free jet region and its environment and the feasibility of capturing ions formed on the impact event using a Waters StepWave ring electrode ion guide. The DMCS study (details in the Supporting Information) revealed that the optimal surface geometry is spherical and its optimal position is just behind the position of the Mach disk formed in the absence of a collision surface. The simulation has revealed that if singly charged 1 kDa particles with a diameter of 2.2 nm are generated on the surface of a spherical collision surface positioned in the Mach disk region then the ion guide of a commercial mass spectrometer can efficiently capture these secondary ions. We constructed a REIMS source with these parameters and coupled it with a spatially resolved laser desorption setup. A two-dimensional automated stage was chosen for the imaging platform, and the stage was combined with dedicated optics setups. Three mid-infrared laser systems were compared to study the effects of different laser ablation parameters and to assess the feasibility of the imaging platform: a surgical CO_2_ laser, a commercially available optical parametric oscillator (OPO), and an experimental optical parametric amplifier (OPA) laser using novel four wave mixing–difference frequency generation (FWM–DFG) technology.^46^ The parameters of the light sources are given in Supporting Information Table S1. The three different laser systems had dedicated focal optic setups for each experimental setup. The CO_2_ laser is delivered by a surgical waveguide, where the beam is first collimated and then focused on the sample. The OPO and OPA systems output a collimated beam, and thus, they require focusing on the sample using only one focusing lens. The systems were utilized for MSI of mouse brain samples to characterize the imaging performance, and then a clinical study was performed on cancerous human breast tissues where the clinical translatability of the method was assessed by cross-validating the MSI results with intraoperative mass spectrometry tools.

**Figure 1:**
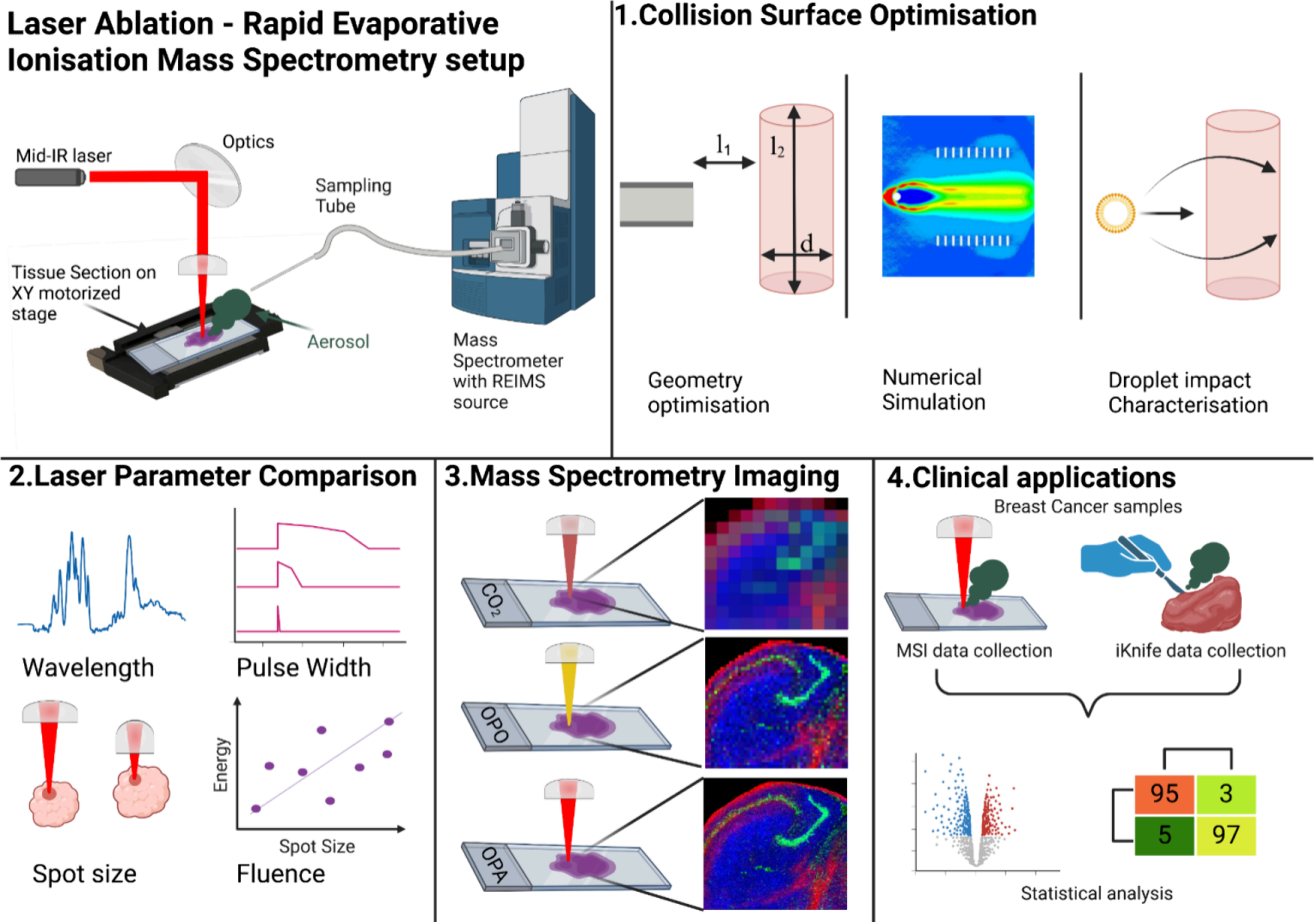
Schematic representation of the LA-REIMS imaging workflow development. The imaging setup was constructed using mid-infrared lasers, laser optics, and an automated XY stage for raster scanning. The imaging platform was coupled with a mass spectrometer equipped with a REIMS ionization source. (1) Numerical simulations were conducted to gain a better understanding of collision surface mechanics and particle behavior around the impact region. (2) Laser parameters were tested and optimized over a broad range of parameters, including wavelength, pulse width, spot size, and fluence. (3) The system was tested for MSI performance and (4) a proof-of-concept study was performed on breast cancer samples to evaluate the clinical translational capabilities of the technique.

### Laser Desorption Method Development

Three different lasers, a commercially available CO_2_, an OPO, and an experimental picosecond OPA, were tested as the primary ablation/desorption tool. Experiments detailed in the “Laser Parameter Characterization and Imaging” section of the Supporting Information document showed that the best signal-to-noise ratio spectra were obtained when using wavelengths close to water absorption maxima points. While the signal-to-noise ratio (SNR) varied between different wavelengths, there were no significant shifts in observed spectral features between different wavelengths. These results suggest that the laser interaction with the tissue is limited to the ablation/mobilization due to the rapid heating of the sample (using a nonresonant laser like CO_2_) or the nonequilibrium elimination of the H-bonding network holding the sample molecules together (in the case of resonant IR lasers), and there are no additional interactions (e.g., photochemical ionization) between the laser radiation and the sampled material. These results fall in line with results obtained by other techniques that also utilize the natural water content to ablate tissues using resonant IR laser desorption.^41,44,48,49^

The pulse width of the different laser systems varies over a wide range, and the effects were tested on mouse brain tissues. The spectral profiles of mouse gray and white matter obtained with the three different lasers are shown in Supporting Information Figure S6A,B. In the case of the CO_2_ laser, the SNR was found to be lower in the complex lipid region (600–1000 *m*/*z*, which has been widely used to establish histological identity) than with the other OPO and OPA sources. These differences are associated with the different rates of heating, which is a consequence of different wavelengths and different pulse durations. The wavelength determines the relative kinetics of sample ablation (disintegration to intact molecules) and heating. The pulse width, on the other hand, determines the level of confinement, a phenomenon observed when laser pulses interact with solid surfaces.^50^ In the case of the long pulse width (100 μs) CO_2_ laser, the ablation is not confined thermally, meaning that the laser irradiation extends well beyond the sample aerosolization,^51^ and the thermally conducted energy causes the thermal degradation of the molecules, which is further exacerbated by the off-resonance ablation. The resulting slow heating of biological macromolecules generally involves condensation reactions via the loss of water and ammonia from hydroxyl and amino moieties (cf. Mallard reaction) yielding a cross-linked covalent matrix eventually turning into amorphous carbon, known as carbonization.^52^ This process effectively shifts the aerosol formation from aqueous droplets toward soot. The downstream declustering process is effective only for liquid droplets, as the energy regime is not sufficient to break up the covalent matrix of soot/carbon particles, resulting in poor sensitivity. Short (5–7 ns) laser pulses emitted by the OPO laser cause negligible thermal degradation of the biological material because most of the laser energy is utilized for the thermally confined ablation/explosion,^53^ which is further enhanced by the resonant wavelength. The picosecond source allows the emission of ultrafast pulses in the range of tens of picoseconds, putting the ablation in the stress-confined regime, which increases the efficiency of ablation, resulting in a higher yield of intact biomolecules (metabolites, phospholipids, other small molecules).^54^ Stress-confined pulses interacting with the tissue cause more efficient energy utilization due to the laser energy being utilized primarily for the ablation process, not for the generation of acoustic shock waves,^53^ explaining the roughly similar signal intensities per pixel with the OPO laser, even though the ablation spot size was significantly smaller (30 μm for OPO, 10 μm for OPA).

### Mass Spectrometry Imaging

Mouse brain mass spectral images were acquired using all lasers (results shown in Supporting Information Figure S6C–E). The data reveals that all modalities can be utilized to acquire information from spatially heterogeneous samples. The spectral profiles mainly consist of small molecules (metabolites, fatty acids, amino acids, and phospholipids), which is in agreement with results obtained previously by iKnife/REIMS.^52^ A list of identified molecules is given in Table S2. The annotation was performed by using accurate mass analysis and the Chemical Abstract Services database. The results were validated on tissue extract analyzed by a well-characterized LC–MS method.^47^ The set of observed molecular species shows a good overlap with species described in the literature on applied laser desorption or REIMS-based MS techniques.^55,56^ The OPA system’s superior beam quality (M^2^ < 1.5) and smaller spot size (10 μm) compared to the OPO (M^2^ > 5, 30 μm spot size) enabled imaging of the hippocampal region at a 10 μm pixel size. The OPA image (Supporting Information Figure S6E) shows the distribution of guanine (150.04 *m*/*z*), a nucleobase that correlates well with the distribution of cell nuclei on the haematoxylin and eosin (H&E) optical microscopy image, allowing the identification of cell nuclei in the mass spectral image. By using oversampling, 5 μm pixel size mouse brain imaging was performed with a modified version of the OPA system,^57^ and the results are shown in Figure 2. The ion images of 134.04 (Adenine [M-H]^−^), 766.54 (PE 38:4), and 955.72 *m*/*z* (unknown) (Figure 2A–C) show highly resolved brain structures in the thalamus region. The optical image after sampling is shown in Figure 2D. A zoomed-in section of the heat map distribution of adenine and the aligned optical region is shown in Figure 2E,F. Notable heat map and optical regions are assigned numbers; these show individual cell nuclei and the associated hotspot distribution of the nucleobase. These results confirm that with proper light source and optical configurations, single-cell resolution imaging is feasible using LA-REIMS.^50,51^

**Figure 2:**
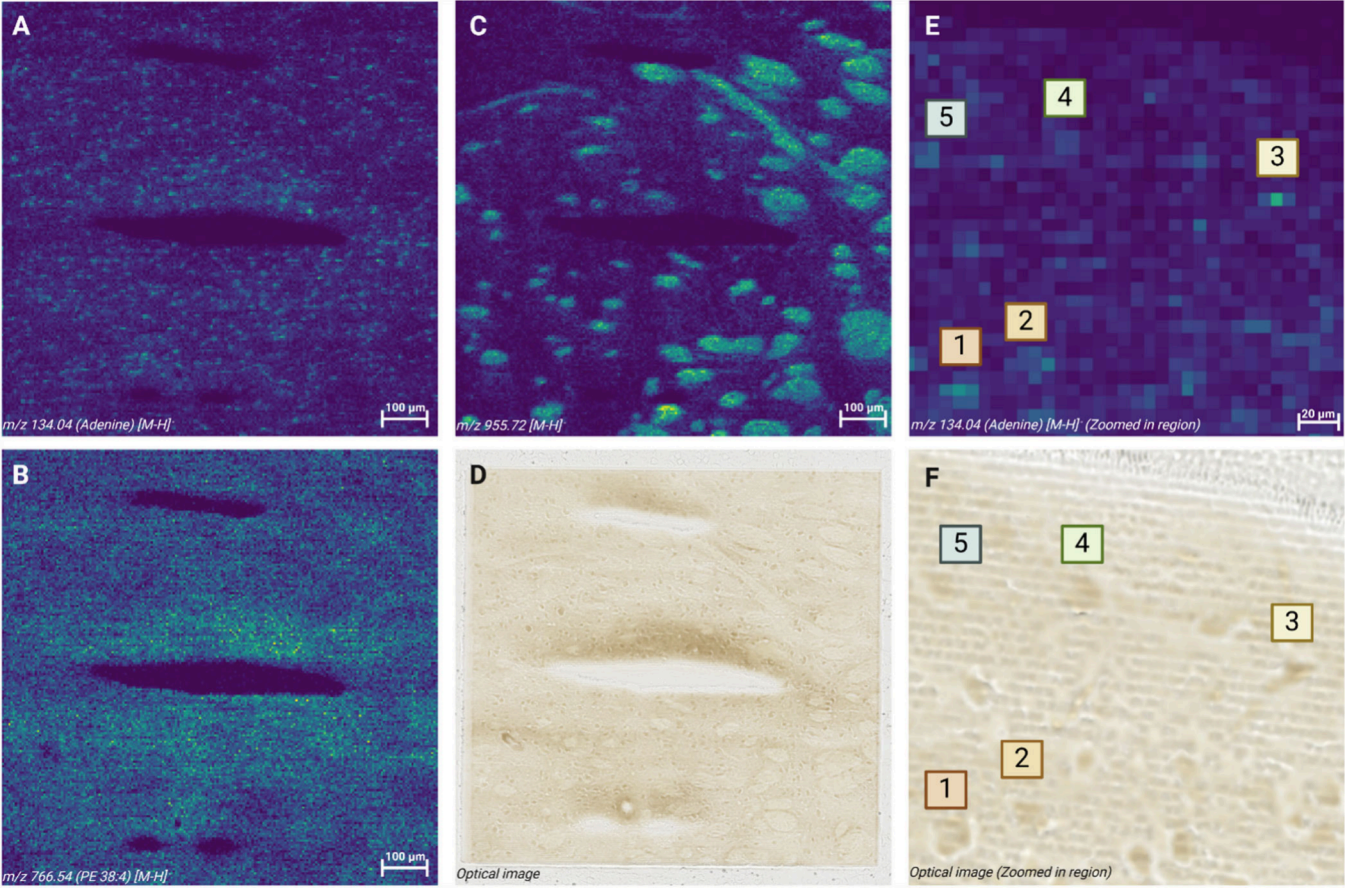
Five micrometer high-resolution imaging using the picosecond LA-REIMS setup. A 1 × 1 mm^2^ area of the thalamus region of a mouse brain was analyzed using the high-resolution picosecond laser setup. The distributions of 134.04 *m*/*z* (Adenine [M-H]-) (A), 766.54 *m*/*z* (PE 38:4 [M-H]-) (B), and 955.72 *m*/*z* (unknown) (C) show high spatial matching with the tissue features observed in the optical image postanalysis (D). Individual cell nuclei were identified with numbers from 1 to 5 on a zoomed-in section of the distribution of adenine (E) and the aligned optical image (F).

### Clinical Application of Mass Spectral Imaging

A clinical case study was performed with the CO_2_ and OPO laser setups, using cancerous human breast tissue samples to demonstrate the translational and molecular pathology capabilities of the system. The OPA laser was excluded from the current clinical study, as this system is available only as an experimental platform, while the other two systems are commercially available sources, with the CO_2_ laser being approved for medical use. Figure 3A shows the intensity of individual significant metabolites and lipids, which also confirms the previous assessment, as the same features observed with both lasers show a clearer distribution using the OPO source compared to the CO_2_ laser. Univariate analysis of the data set confirms that the number of significant features observed was larger with the OPO data (1082 features) versus the data obtained with the CO_2_ laser (286 features), visualized by the volcano plot shown in Figure 3B,C. The MSI results of the cancerous breast tissues in Figure 2D–G and H–K and the correlations to the gold standard H&E staining method (Figure 3D,H) were evaluated using hyperspectral correlation methods.^58^ The characteristic differences in molecular profiles found in the imaging data show good correlation with the pathologist-annotated histologically separated tissue regions (cancerous vs healthy vs fibrous). The observed molecules could enable the method to be used for providing valuable insight into clinically important questions, such as differentiating between breast fibroadenoma and cancer tissues.

**Figure 3:**
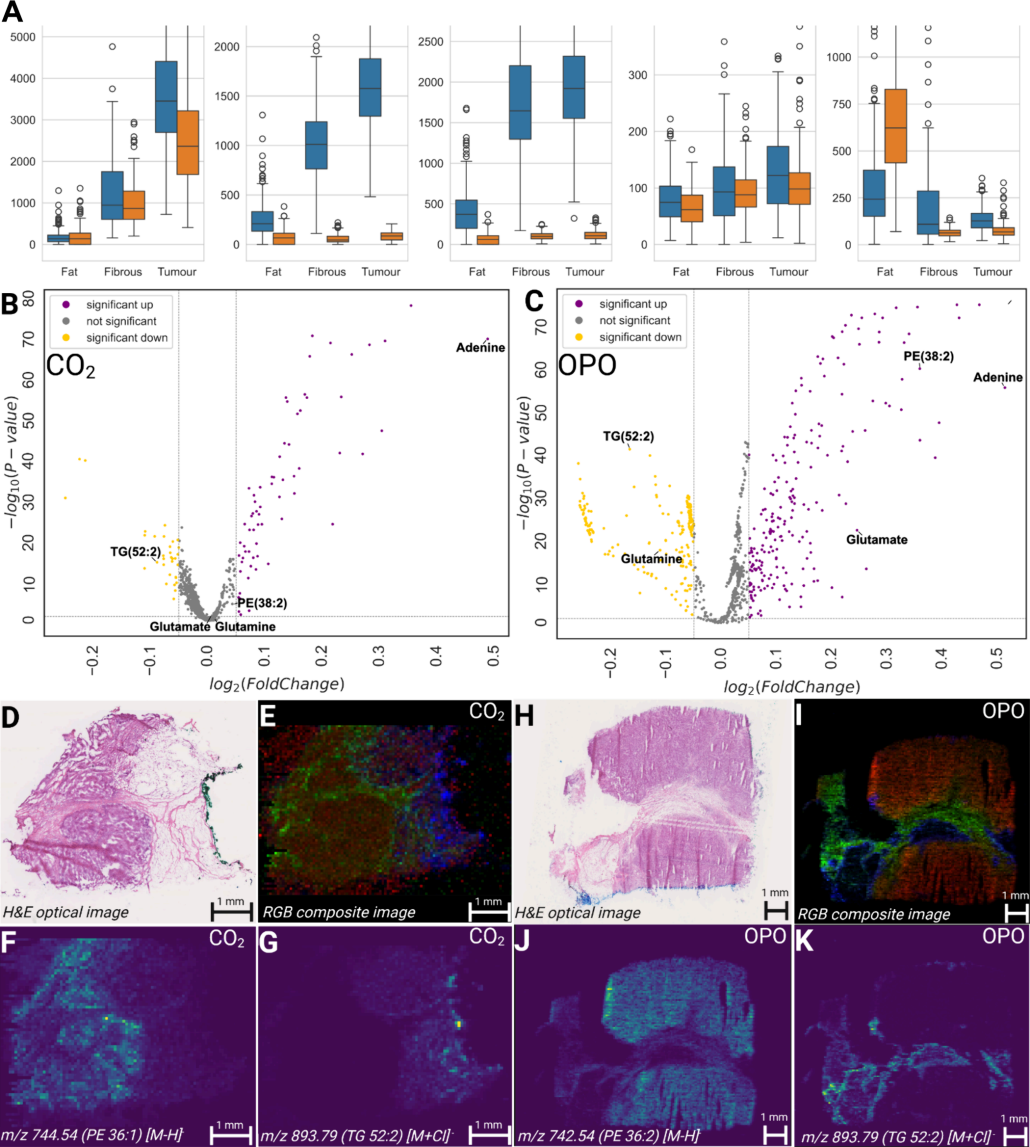
Lipidomic and metabolomic characterization of breast cancer samples using LA-REIMS. Ex vivo human breast samples were analyzed with two different IR lasers using the LA-REIMS method. A: Univariate plots of identified molecules of adenine [M – H]^−^, glutamine [M – H]^−^, glutamate [M – H]^−^, phosphatidylethanolamine PE(36:2) [M – H]^−^, and triglyceride TG(52:2) [M + Cl]^−^ show significant differences between different tissue types. *Statistically significant difference between fat and fibrous tissue. **Statistically significant difference between tumor and fat tissue. ***Statistically significant difference between tumor and fibrous tissue. The tissues contained healthy and cancerous sections, validated by histopathology analysis. Volcano plots generated from the data obtained with the two lasers (B, C) allow us to identify numerous metabolites and phospholipids that show statistically significant fold changes. The breast cancer samples were analyzed using the two lasers at 70 μm raster sizes. Consecutive tissue sections were H&E stained and classified by pathologists (M-CO_2_ and Q-OPO). The results of the CO_2_ laser can be seen in panels D–G, where the composite image (E) is visualized (red: 744.54 (PE 36:1 [M – H]^−^); green: 217.05 *m*/*z* (unknown); and blue: 893.79 *m*/*z* (TG 52:2 [M + Cl]^−^)). The results of the OPO imaging can be seen in panels B–E, where the composite RBG image (B) is visualized (red: 744.54 *m*/*z* (PE 36:1 [M – H]^−^); green: 195.05 *m*/*z* (unknown); and blue: 893.79 *m*/*z* (TG 52:2 [M + Cl]^−^)). Individual ion images for different ionic species (O: 744.54 *m*/*z* and P: 893.79 *m*/*z* for CO_2_; S: 744.54 *m*/*z* and T: 893.59 *m*/*z* for OPO) show good differentiation between different histological status tissues.

Beyond the advantages for high-resolution ambient ionization imaging, the technology also has the potential to bridge MSI with intraoperative mass spectral tissue identification.^52,59^ A proof-of-concept study was conducted where a database and a multivariate model were constructed using the imaging CO_2_ setup and were tested by the iKnife instrument^25^ as a potential training set of the multivariate statistical model used for tissue recognition. For laser sampling of fresh ex vivo specimens and tissues in vivo in the operating theater, the surgical CO_2_ laser was chosen as a device approved for medical use. The spectral profiles from the iKnife utilizing a diathermy tool for REIMS analysis and laser-ablation REIMS data are shown in Supporting Information Figure 9A–F. The multivariate model trained to differentiate healthy vs cancerous tissues using LA-REIMS showed good sensitivity (93.65%) and specificity (97.12%). While the PCA model in Figure 4A,B does not reveal clear visual separation due to the limited number of available tissue data points across both modalities (n = 9), the cross-validation results show that a model built of the laser imaging data was successfully utilized for histopathological classification. Diathermy data classified using a PCA-LDA model built of laser imaging data had 100% correct classification for normal tissue and 92% correct classification for cancerous tissue. The laser imaging data classified using the diathermy data-based model gave 97% correct classification for normal tissue and 100% correct classification for cancerous tissue. These results show that these models can be used across different ablation modalities, raising the possibility of creating method-independent models universally applicable across all surgical energy devices and the corresponding histological MSI modalities.

**Figure 4:**
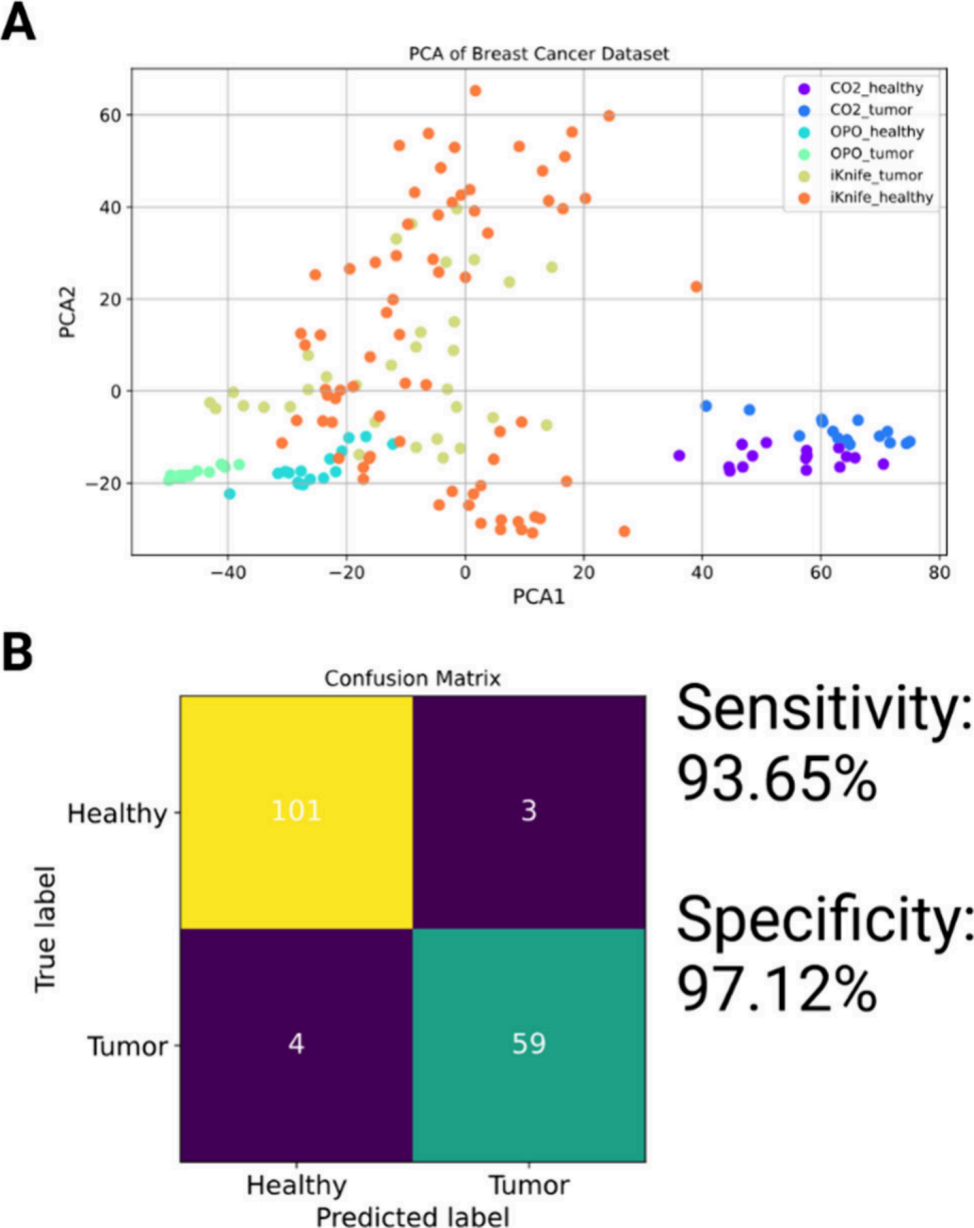
A PCA model was built from breast healthy and tumor data obtained with all ablation modalities for tissue classification (A). Leave-20%-out cross-validation was performed from the previously built PCA-LDA model; the model yielded good sensitivity (93.65%) and specificity (97.12%) (B).

With the data obtained from these experiments, we successfully achieved our goals: 1) understand and optimize REIMS parameters for infrared laser ablation as a sample mobilization method; 2) demonstrate the feasibility of histologically relevant mass spectrometric imaging using the LA-REIMS platform; and 3) demonstrate the feasibility of a “From MSI – to surgical theater” workflow which is enabled by the versatility of the REIMS ionization methodology applicable to both single-cell MSI and to surgical intervention. Numerous mass spectrometry methodologies have been successfully utilized for achieving one or more goals; however, to our knowledge, no MS method has demonstrated the utility of REIMS imaging to create training data for REIMS-guided surgery.

Our long-term goal is to develop a tissue identification system, which can equally be used for chemical histology (i.e., the identification of cells in histological sections) and in a surgical environment for real-time decision making. Current histology techniques are all based on the staining and microscopy of stained sections, which cannot be implemented directly in vivo, leaving surgeons with the option of so-called frozen section histology (FSH),^60^ taking 20–30 min for a single specimen with results loosely correlated with the actual surgical landscape. Similarly, current MSI technologies including MALDI, DESI, SIMS, and other approaches also require histological sections, and although they eliminate the staining steps, they are replaced by matrix deposition (in the case of MALDI) or just lengthy acquisition times resulting in turnaround times very similar to FSH.^61^ In contrast, intraoperative MS techniques (MasSpec Pen, iKnife, PIRL-DIVE, or the closely related SpiderMass technique) are excellent tools for identifying tissues (especially cancerous tissues) in situ and in real time in the medical interventional environment, but they are suboptimal choices for histological analysis. The intended applications for these techniques are similar (surgical *in vivo* mass spectrometry-based tissue classification) but the ionization mechanisms are different: the MassSpec pen uses a water-based liquid extraction–electrospray ionization method, Spidermass uses water-assisted laser desorption ionization, PIRL-DIVE uses an ultrafast pulsed resonant IR LDI process, and iKnife uses REIMS.^25,31,38,39,59,62^ MSI using these techniques has been demonstrated,^63^ but the reported spatial resolutions fell 3 orders of magnitude short of that of standard histopathology. One of the main motivations regarding the development of a universal method is to make intraoperative MS methods provide strictly histology-level information. While the MSI community believes that the metabolic profile of tissues carries more diagnostic and prognostic information than the morphology accessed by standard histology approaches,^64–66^ this MS data currently cannot be interpreted in the absence of properly annotated and validated data sets. MSI data sets available worldwide include specimens obtained from a few thousand patients, most of them in the last 10 years.^67^ In contrast, more than one hundred million histology sections collected in the last 200 years are available, with most of them linked to full clinical histories.^68^ In order to cross-correlate MS profiling data with this vast and unparalleled resource, we need techniques that, besides in vivo analysis, are also capable of single cell resolution analysis of histological sections. This capability will also enable the development of significantly more accurate identification models. Currently, the classification models used in MS-guided surgery are based on the analysis of ex vivo, bulk surgical specimens using intrasurgical sampling tools.^25,29,69^ Consequently, both these and the intrasurgical data points comprise information that originated from tens to hundreds of thousands of cells. The annotation of these sampling points is also not straightforward as the sampled cells are either absent (as they were ablated) or damaged by the sampling procedure.^70^ As a result, all data points correspond to mixed histology with unknown contributions from the individual cell types. A method with single cell analysis capability can resolve this problem by providing clean data for algorithms taking this mixed nature of the intraoperative data into account and providing results with histological accuracy. The methodology demonstrated here does fulfill the criteria; however, we perceive it as a proof of concept, which will undergo detailed validation in the future before it can be clinically tested and deployed. Additionally, the use of identical approaches in the pathology laboratory and in an interventional environment may also draw some attention with regard to clinical data integrity and quality assurance. Furthermore, we have recently demonstrated the utility of LA-REIMS for the detection of microorganisms.^71,72^ The combination of the three applications (surgery, histology, and microbiology) enables the development of a new tissue analysis tool with unprecedented versatility for diagnosing and treating a wide variety of diseases ranging from cancer to autoimmune and metabolic disorders to infections and dysbiosis.

## Conclusions

In the study, the concept of a sample-preparation-free, “from histology to surgery” molecular workflow was successfully developed using laser ablation-based sample mobilization coupled with rapid evaporative ionization mass spectrometry-based impact declustering ionization. Using numerical simulation methods, the impact declustering parameters were optimized to maximize the sensitivity of laser ablation-REIMS. The optimal collision surface position was found to be 5 mm from the inlet capillary, and the ideal temperature was determined to be approximately 1000 K. Three different laser sources—a CO_2_, an OPO, and an experimental OPA laser operating at microsecond, 5–7 ns, and 100 ps pulse widths—were utilized for 70 to 5 μm resolution tissue imaging, successfully demonstrating single-cell resolution capabilities under ambient conditions. We also demonstrated in a proof-of-concept study (n = 9) the feasibility of utilizing the histologically annotated imaging data set for classifying spectra acquired under conditions analogous to those used in a surgical operation (through diathermy and the iKnife application) with high specificity and sensitivity (93.65 and 97.12%, respectively). The presented results serve as a basis for future work regarding sample-preparation-free molecular imaging of clinically important tissues and other samples. Since the technique requires no labeling, sample preparation, or other user interaction with the process, the method has a high potential for nontargeted, automated molecular profiling of human disease samples.

## Supplementary Material



## Notes

Numerical simulation optimization workflow; experimental data (spectral profiles) on laser comparison; imaging performance translational spectra; tables of laser parameters; and list of annotated metabolite features (PDF)
