# Supplementary material for: Subcellular-Resolution Molecular Pathology by Laser Ablation–Rapid Evaporative Ionization Mass Spectrometry
Source: Anal Chem. 2025 Aug 6;97(32):17433–43. doi: 10.1021/acs.analchem.5c02013 (PMC12368837; doi:10.1021/acs.analchem.5c02013)
Supplement: Supplementary file 1 [file ac5c02013_si_001.pdf]

Subcellular Resolution Molecular Pathology by Laser Ablation – Rapid Evaporative Ionization Mass Spectrometry

Dániel Simon [a,b,c], Gabriel Stefan Horkovics-Kováts [d,e], Yuchen Xiang [a], Ronan A. Battle [f], Yu Wang [f], Julia Abda [a], Dimitris Papanastasiou [g], Stefania Maneta Stavrakaki [a], Hui-Yu Ho [a,h], Haixing Wang [a], Richard Schäffer [e], Tamás Karancsi [e], Anna Mroz [a], István Pap [e], Laurine Lagache [i], Júlia Balog [e], Isabelle Fournier [i], Robert T. Murray [f], Josephine Bunch [j], Zoltan Takáts [a,b,c,i*]

Corresponding Author

[a] Department of Metabolism, Digestion and Reproduction, Imperial College London Exhibition Road, London, SW7 2AZ, United Kingdom

[b] Rosalind Franklin Institute, Fermi Avenue, Didcot, OX11 0QS, United Kingdom

[c] Institut für Funktionelle Genomik, University Regensburg, 9 Am Biopark, Regensburg, 93053, Germany

[d] Hevesy György PhD School of Chemistry, ELTE Eötvös Loránd University 1/A Pázmány Péter sétány, Budapest, 1117, Hungary

[e] Waters Research Center, 7 Zahony utca, Budapest, 1031, Hungary

[f] of Physics, Imperial College London, Exhibition Road, London, SW7 2AZ, United Kingdom

[g] Fasmatech, TESPA Lefkippos, NCSR Demokritos, Athens, 15310, Greece

[h] Department of General Surgery, Linkou Chang Gung Memorial Hospital, No. 5, Fuxing St, Guishan District, Taoyuan City, 333, Taiwan

[i] PRISM Inserm U1192, University of Lille, 42 Rue Paul Duez Lille, 59000, France

[j] National Physical Laboratory, Hampton Road, London, TW11 0LW, United Kingdom

[*] Email: z.takats@imperial.ac.uk

ABSTRACT: This work demonstrates the combination of ambient laser ablation (LA) with in-source surface-induced declustering, originally developed for Rapid Evaporative Ionisation Mass Spectrometry (REIMS). This combination, termed laser ablation REIMS (LA-REIMS), provides sensitivity, spatial resolution and chemical coverage comparable to Matrix Assisted Laser Desoprtion Ionisation (MALDI), but without the requirement for matrix deposition. The atmospheric pressure interface setup was subjected to detailed characterization with regard to geometric and thermal parameters, augmented by in-silico flow modeling. The resulting platform was tested using aerosol formed by infrared laser ablation of tissues. Three different laser systems were successfully employed for ambient mass spectrometric imaging: a carbon dioxide laser (λ=10.6 µm, τ_L=~100µs), an optical parametric oscillator (OPO; λ=2.94 µm, τ_L=8 ns) and an optical parametric amplifier (OPA; λ=3.0 µm, τ_L=~30 ps). Single-cell imaging was achieved using the OPA systems high-resolving capabilities, metabolites and lipids ranging from amino acids through carbohydrates and nuclear bases to complex glycolipids were successfully detected. The technique was also tested as a platform for MS-guided surgery, raising the possibility of using a single technique for generating histological and in-vivo data

Supporting Information

Contents

Numerical simulation and Optimization of Surface-induced Declustering in the Atmospheric Interface S2

Laser Ablation fundamentals and imaging workflow optimisation S7

MALDI comparison S12

Supplementary tables S13

Table S1: Properties and operation mode of the different lasers used for the experiments. S13

Table S2.: List of annotated peaks observed during the imaging experiments in mouse brain sections. S17

Table S3.: List of lenses and measured power values used for the fluence experiments. S18

References S19

Numerical simulation and Optimization of Surface-induced Declustering in the Atmospheric Interface

Characterization of the flow structure was performed sequentially using different solvers and tools to cover the wide pressure range of the flow field and perform particle tracing respectively. The first step involved the application of the Reynolds-Averaged Navier-Stokes method to solve the high-pressure region inside a 70 mm, 0.5 mm I.D. inlet capillary at room temperature. A structured high-density mesh was generated to describe the laminar flow across the capillary. Zero slip velocity was imposed on the inner walls of the capillary. Mass averaged velocity, temperature and gas density values were determined at 0.2 mm from the capillary exit as 335 m/s, 240 K and 243 mbar and subsequently imposed as inlet boundary conditions for the Direct Simulation Monte Carlo (DSMC) calculations. The DSMC domain was constructed with a 5 µm cell size progressively increased to 10 µm in regions distant from the impingement target to increase computational speed without affecting the accuracy of the solution near the target. The background pressure was 3 mbar. DSMC calculations were performed in the low-pressure region of the domain using the SPARTA solver. The flow field solutions were then further processed in MATLAB and introduced into SIMION for tracing particles. A thousand 100 kDa neutral particles with 10.2 nm diameter were used to simulate solvent clusters. Their position and impact velocity were recorded and then used to generate an ion cloud of 1 kDa particles with 2.2 nm diameter. A geometric representation of the ion source region was generated (illustrated in Supplementary figure S1A) and different shaped and sized collision surfaces were inserted in different geometric configurations.

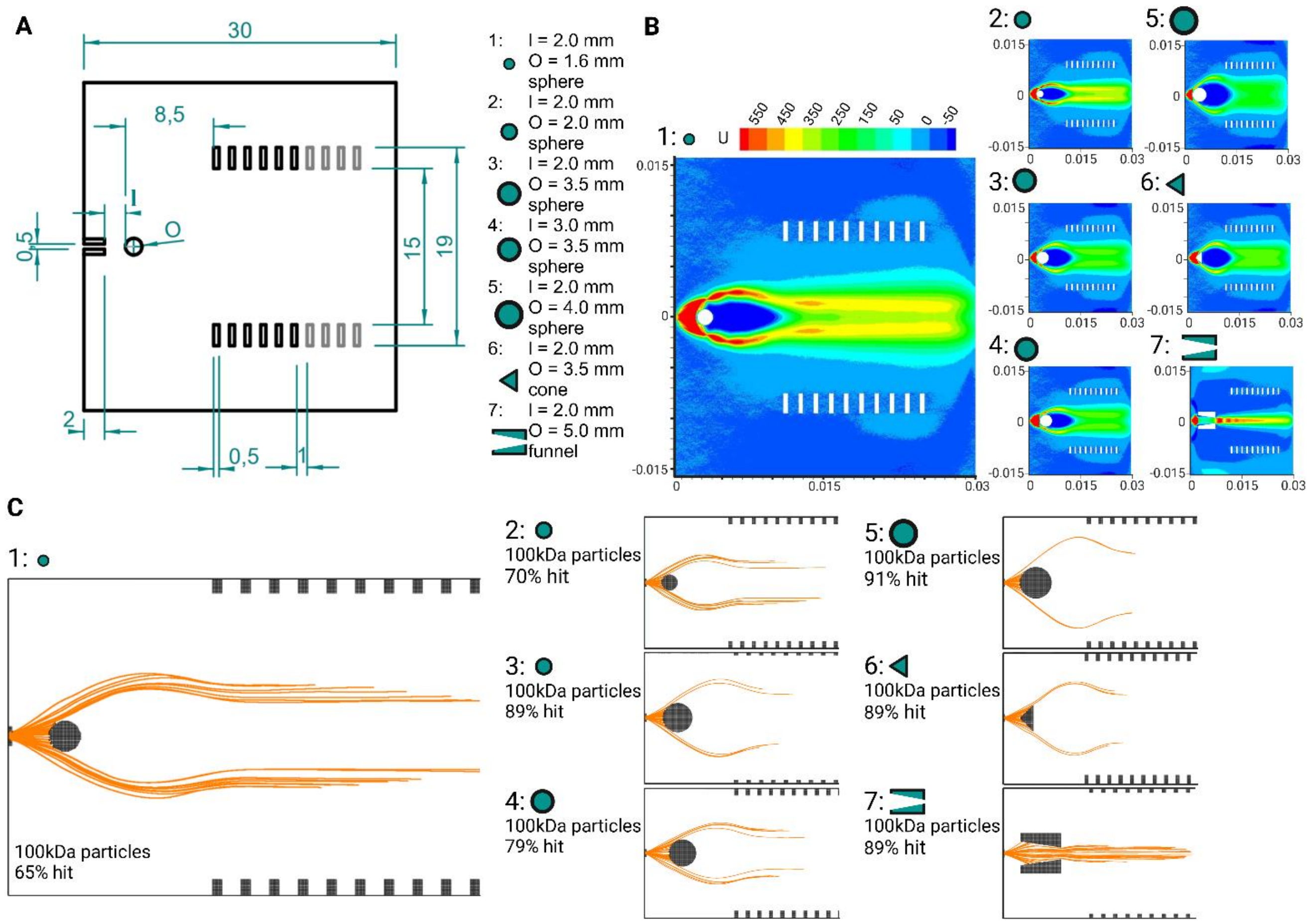

Supplementary Figure S 1: Optimisation of the impactor surface using DSMC numerical simulations.

The prototype atmospheric interface design allowed the rapid and efficient experimental characterisation of different collision surface geometries in addition to the numerical simulation data. DSMC simulations were performed in the modelled atmospheric interface environment (A) using the following geometries: 1.6 mm sphere, 2 mm distance from inlet capillary; 2 mm sphere, 2 mm distance from the inlet; 3.5 mm sphere, 2 mm from inlet; 3.5 mm sphere, 3 mm from inlet capillary; 4 mm sphere, 2 mm from the inlet; 3.5 mm cone, 2 mm from inlet capillary; 3-1.5 mm decreasing funnel, 5 mm long, 2 mm from inlet capillary . The axial velocity profiles (B) were characterised, and 100k amu particle collision simulations (C) were performed.

The focus of the declustering modelling part of the work is to describe the behavior of aerosol particles in the presence of an impactor surface and provide an explanation for the impact-based declustering post-ionization phenomena, of which understanding will be critical for assessing the robustness and sensitivity of the technology. The primary particle trajectory simulations (Supplementary Figure S1B) revealed that the majority of particles entering the intermediate vacuum regime of the mass spectrometer (where the impactor surface is positioned) impact on the jet disruptor surface at an average impact velocity of approximately 600 m/s. The specific kinetic energy of the particles (100kDa, 10.2 nm diameter) is very low – it is ~2 eV/kDa -, insufficient to break covalent bonds, but certainly sufficient to breakup molecular clusters (albeit insufficient for their complete evaporation, cf. the vaporization enthalpy for isopropanol is 45.48 kJ/mol). The trajectory simulations for secondary particles in Supplementary Figure S1C clearly showed that even individual molecular ions of complex lipids (represented by 1kDa) particles are readily entering the travelling wave ring electrode ion conduit. Five different impactor geometries were modelled, including spheres with different diameters (2 – 4 mm), cones, conical apertures and cylinders, at set distances from the inlet capillary. The numerical simulations show that the spherical collision surface performed the best regarding impact kinetics (the particles achieving the highest velocity prior to impact on the surface). This distance was experimentally determined to be 5 mm from the capillary inlet. The optimal geometrical configuration with current inlet capillary (0.5 mm I.D.) and traveling wave ring electrode setup thus was determined to be a 3 mm diameter spherical collision surface positioned 5 mm from the MS transfer capillary. Experimental data shows an optimal intensity for the total ion count (supplementary figure S2A-D) and for example biomolecule species (palmitic acid – 1B, Phosphatidylethanolamine PE(36:2) – 1C and Phosphatidylethanolamine PE(38:4) – 1D) at 5 mm capillary distance from the collision surface. This geometric setup allows the free jet expansion and acceleration of the sample droplets which collide with the optimally located impactor surface for maximum ionization efficiency. The optimal distance shows a good correlation with the position of the Mach-disk region (the spatial region where the supersonic jet collapses[1]) of the free jet. The velocity and the kinetic energy of the individual particles is maximum before the Mach-disk, thus the positioning of the impactor surface at this region explains the highest ion yields obtained by the high-velocity impact at the surface.

The atmospheric interface setup was tested using continuous aerosol introduction. In order to check the consistency of experimental observations with the DMCS predictions, signal intensity was tested as the function of the longitudinal position of the collision surface. The experimental data shown on Figure S2 shows excellent agreement with the prediction. Although the introduction of a solid collision target at the optimal position resulted in a dramatic improvement in signal intensity (compared to no collision surface), the time-dependent signal intensity in this case also showed a remarkable decline in the timescale of minutes. We were able to recover full signal intensity by thoroughly cleaning the surface, however regular cleaning after a few 10 minutes of use was deemed to be incompatible with the envisioned biomedical applications. Replacing the ambient temperature spherical impactor with a Kanthal D coil (made of 1 mm ribbon, coiled in a 3 mm diameter coil, 5 mm long) (Supplementary Figure S2E-F) surface with controlled temperature in the range of ambient to 1400K alleviated these problems. Although the coil (or band) does not provide a well-characterised surface geometry, any coating (e.g. a ceramic tube) results in a substantial drop in the temperature of the surface at the collision point due to the cooling effect of the free jet expansion. Experiments show that heating the surface of the impactor to around 1200K dramatically increased the signal intensity and decreased memory/carryover issues, caused by the buildup of material on the impactor surface, as seen in Supplementary Figure S2E-I. While the absolute optimum might be higher than 1200 K, the proximity of the high temperature collision surface to the ion guide resulted in adverse effects above this temperature including discharge and deformation of the ion guide. Besides providing a stable signal for several hours, the heated surface increased the signal in tensity by a further three orders of magnitude (supplementary figure S2E-I) compared to the cold surface from pork liver samples. The elevated temperature of the impactor enhances the declustering and provides additional thermal energy to complete the subsequent declustering process to yield individual molecular ions for analysis in the mass spectrometer. Note that the kinetic energy of the clusters is insufficient for their complete evaporation even assuming completely inelastic collisions (≈12 kJ/mol vs 45.48 kJ/mol heat of evaporation), therefore any heat exchange with the surface is expected to enhance the process. This effect plateaus at around (possibly above) 1200K, in agreement with the assumption that maximum ionisation efficiency is achieved when clusters completely evaporate on collision. The elimination of memory effects and increase in sensitivity allowed us to utilize the laser desorption method for imaging experiments which require instrument stability over extended periods of measurement time. While the exposure of analyte-containing aerosol particles to 1200 K will certainly result in massive thermal degradation, these effects were not observed, not even when the surface temperature temporarily reached 1400K. The lack of thermal degradation was associated with multiple factors counteracting this effect. These factors include the kinetic shift, i.e. the very short time the molecules spend at this high temperature, their originally very low temperature caused by the expansion and a potential Leidenfrost-like effect, when the droplets approaching the surface start vigorously boiling on their side facing the hot surface and the nascent vapour avoids direct contact.

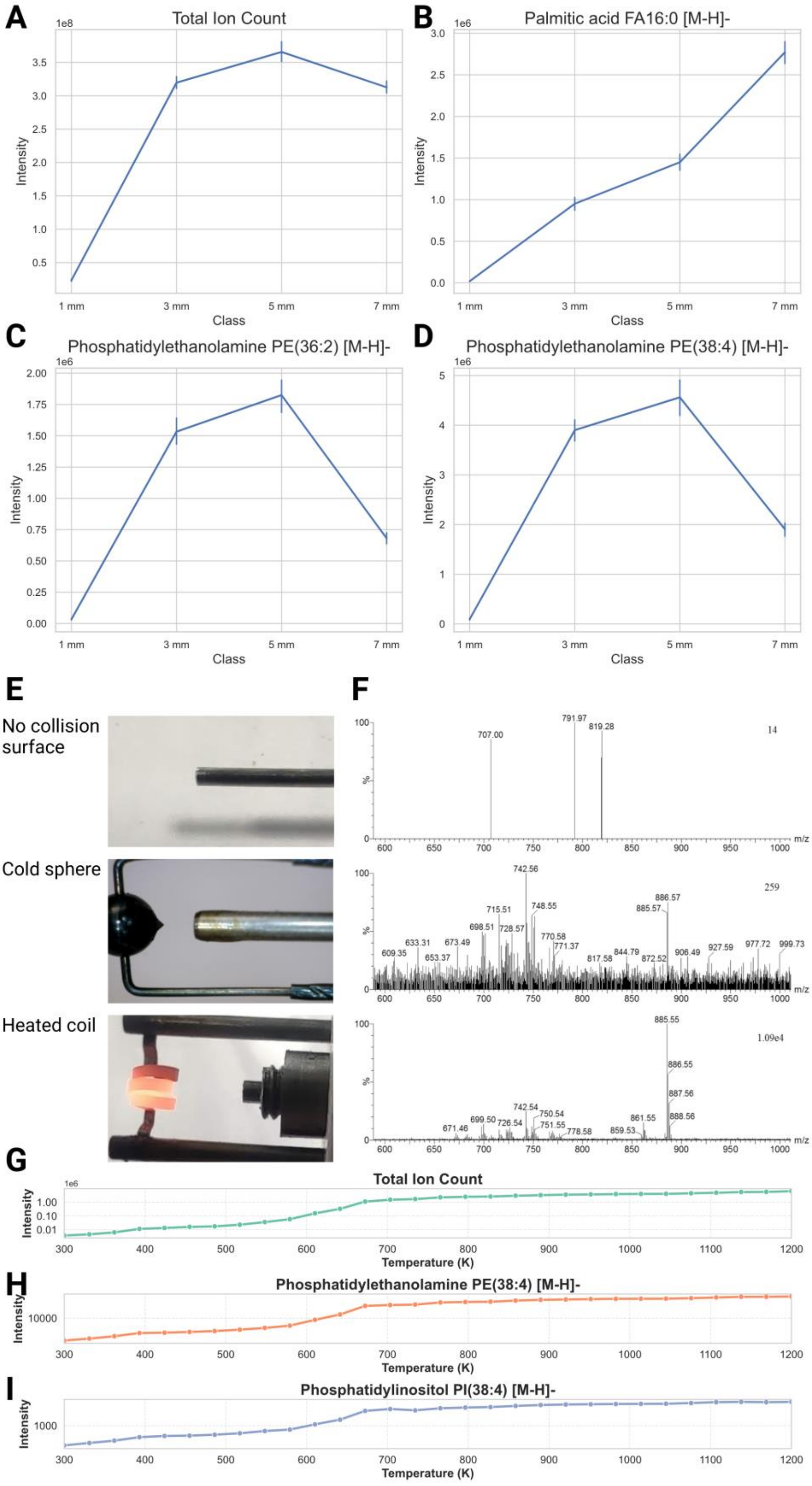

Supplementary Figure S 2: Experimental data on the collision surface optimisation. Effect of the collision surface – capillary distance on Total Ion Count (A), palmitic acid [M-H]- (B), Phosphatidylethanolamine PE 36:2 [M-H]- (C) and Phosphatidylethanolamine PE 38:4 [M-H]- (D). The optimal ion intensity is at 5 mm capillary distance. The effects of the presence of a collision surface and the temperature can be seen in E and F. The effect of heating from 300 - 1200K on the total ion count and PE 38:4 and phosphatidylinositol PE 36:2 can be seen in figures G-I.

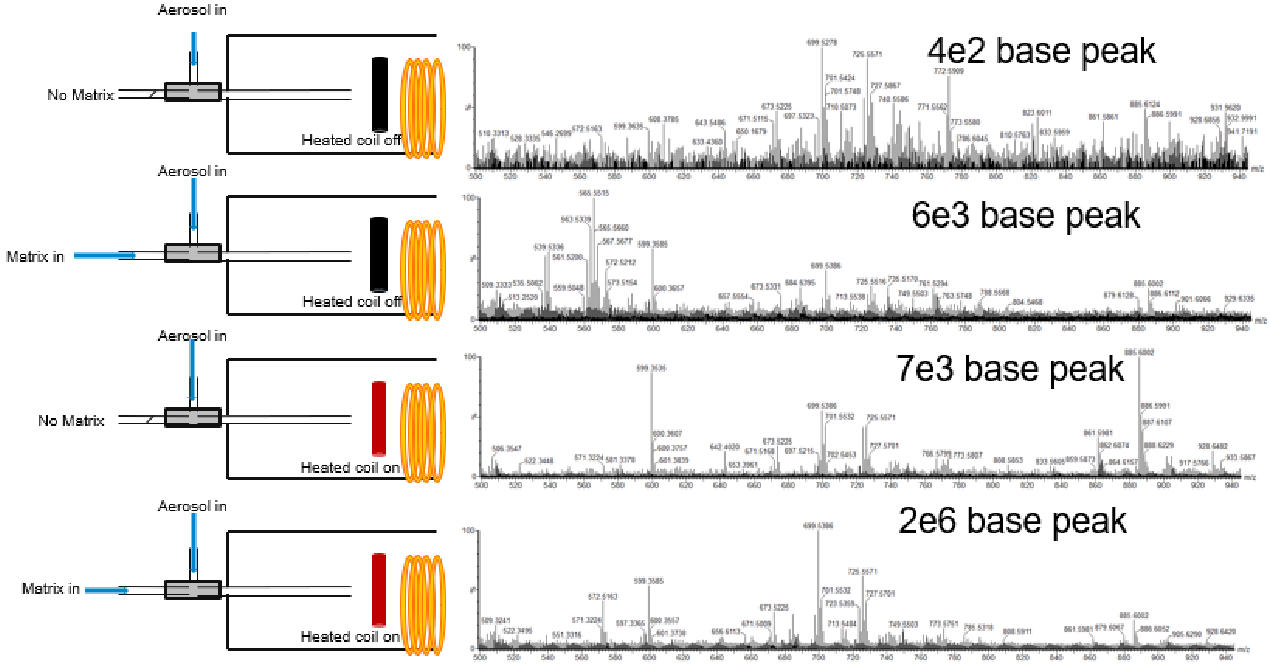

Supplementary Figure S 3: Experimental results from OPO laser using different source inlet settings. Using the OPO laser and porcine liver tissue, four different experimental configurations were tested: No matrix and no heating on the coil; matrix (isopropanol) and no heating; no matrix and heating and both matrix and heating utilised. While spectral features are observable under all configurations, the sensitivity is increased by three orders of magnitude when both the matrix and heating are applied to the samples. This increase in sensitivity enables system to be utilised for single cell/small volume experiments.

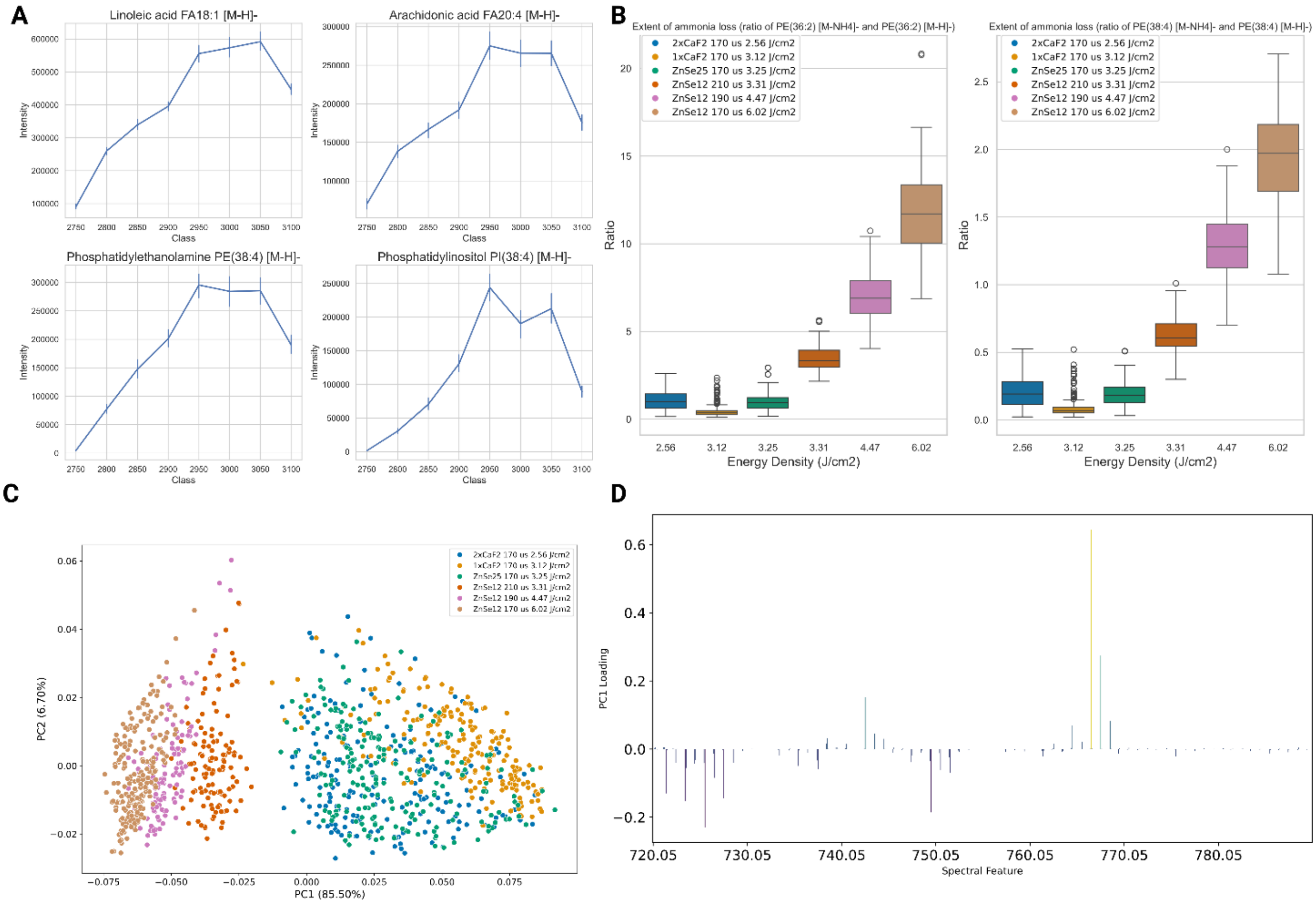

Supplementary Figure S 4: Effects of laser wavelength and fluence on the spectral composition and molecule fragmentation. The tuneable OPO laser allowed the characterisation of the effect of wavelength on the desorption process on pork liver tissue. The available wavelength range (2700 – 3100 nm) was scanned in 50nm resolution; the laser energy was normalised to provide the same fluence for each wavelength. Spectral profiles were recorded in the range of 2750 – 3100, 2700 nm provided no observable spectra

due to low laser absorption rates. The intensity profile of four known molecules over the tested range (FA 18:1, FA 20:4, PE 38:4 and PI 38:4) (A) show an optimal desorption wavelength at around 2900 – 3000 nm. This falls in line with the water absorption maxima observed at 2940 nm in the mid-IR range originating from the O – H bond stretch absorption. The loss of ammonia from phosphatidylethanolamine lipids can be observed with the LD REIMS process. The ratio of observed ions of two PE species (PE(36:2) and PE(38:4)) originating from pork liver samples is shown to increase as the laser fluence increases (B). The PCA model can distinguish between spectra acquired at different fluence levels, as seen in plots C and D. The deamination process of PE species becomes prevalent with laser fluence levels above 3.3 J/cm^2.

Laser Ablation fundamentals and imaging workflow optimisation

Following optimization, the ablation spot size diameters achieved for the setups were 70 µm for the CO_2 laser, 30 µm for the OPO laser and 10 µm for the OPA laser. At the ablation spot, the laser power density of the CO_2 laser was 1 kW/cm^2, 12.55 J/cm^2. The OPO laser fluence was 5 J/cm^2, and the OPA was 0.157 J/cm^2. The mass spectrometer's sampling frequency for the imaging experiments was set to 10 Hz as a good compromise between speed and sensitivity, due to the limited output frequency of the OPO laser and the acquisition parameters of the mass spectrometer. The effects of wavelength tuning were tested using the tuneable OPO source in the available wavelength range (between 2700 – 3100 nm) using pork liver samples while the fluence was normalized to 5 J/cm2. From the mass spectral data, four mass bins (279.25, 303.25, 766.55 and 885.55 m/z, assigned as linoleic acid FA18:1 [M-H]$^-$; arachidonic acid FA20:4 [M-H]$^-$; phosphatidylethanolamine PE(38:4) [M-H]$^-$ and phosphatidylinositol PI(38:4) [M-H]$^-$) were selected to demonstrate the effects of wavelength, shown in Supplementary Figure S3A. Results show that there is an optimal wavelength between 2950 – 3000 nm for intensities of the demonstrated molecular species, which wavelength range is in close proximity to 2940 nm[39], which corresponds to the absorption maximum for the O-H bond stretch band in water. To characterize the spectral pattern-level changes associated with different wavelengths, multivariate statistical analysis (Principal Component Analysis – PCA) was performed on the data. The two principal component (PC) axes visualized on Supplementary Figure S4 (combined PC1-PC2 score: 93.7%) show no significant separation or differences among data points, except from the data obtained at 2750 nm. The signal-to-noise ratio (SNR) for the 2750 nm measurement point was significantly lower (due to the decreased absorption of the O-H stretch bond, thus reduced ablation efficiency) than at other wavelengths, resulting in the separation from the rest of the group.

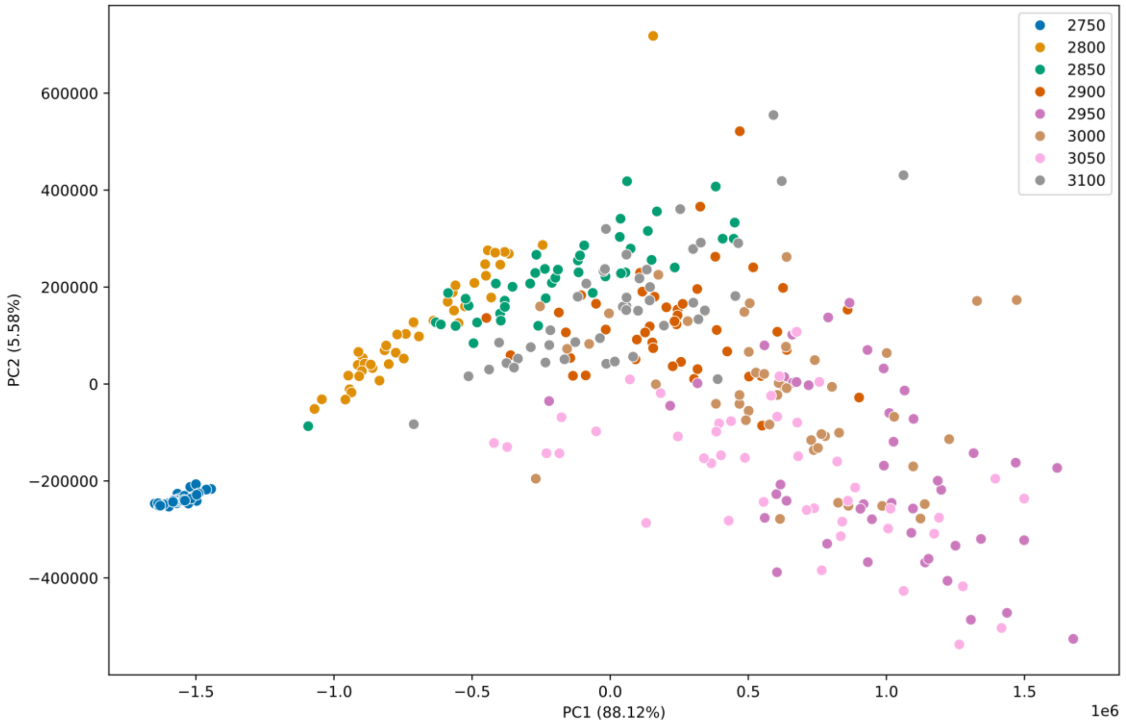

Supplementary Figure S 5: Visualization of the first two Principal Components of the wavelength data. The plot shows no significant separation between wavelengths except the 2750 nm data, which is separated due to the low Signal-to-Noise ratio.

The effect of laser fluence was tested using different focal length lenses and the laser operated at different power levels. The best SNR was achieved with the highest laser fluence using the shorter focal ZnSe lens (at 6.02 J/cm^2), and a characteristic ammonia loss (SN2-like deamination facilitated by phosphate oxygen, previously observed with other applications of the REIMS technique[2]) was observed within the phospholipid region in the spectra, specifically in the case of phosphatidylethanolamine (PE) species. The ratio of observed $[M-NH_4]^-$ to $[M-H]^-$ ions gradually shifted towards a more prominent ammonia loss for PE molecules, as can be seen in Supplementary Figure S3B. A PCA model was also generated using this dataset, where the most prominent differentiating factor in PC1 (85.5%) was observed to be the ammonia loss. Significant separation was observed in spectra obtained between fluences above and below 4.47 J/cm^2 which separation is primarily driven by the ratio changes of deaminated and deprotonated peaks of the same lipid species. The loading and the PCA models are shown in Supplementary Figure S3C-D. Higher energy ablation caused a more stable and reproducible ammonia loss effect, which improved the reproducibility and robustness of the technique overall. The ammonia loss from PE species is also observable with the CO_2 laser at all energy levels due to the long pulsed (>100 microsecond) laser not being thermally confined. Based on these results the minimal ablation threshold for LA-REIMS with the OPO laser setup was determined to be at 4.5 J/cm^2, and laser fluence of 5 J/cm^2 was chosen for the imaging experiments.

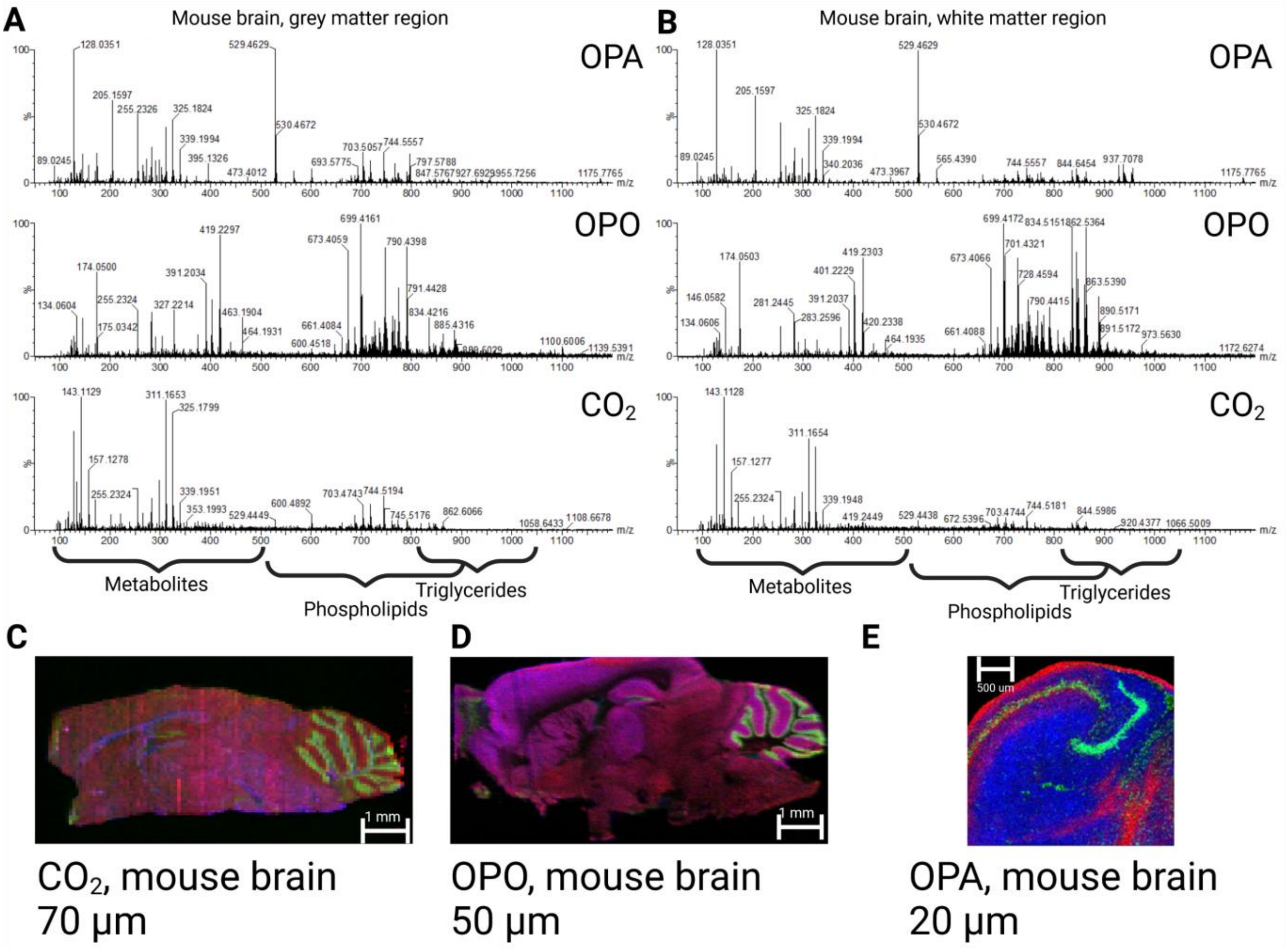

Supplementary Figure S 6: Data comparison between the three different lasers using mouse brain tissues. Spectra obtained from mouse brain grey matter (A) and white matter (B) show significant differences in the metabolic and lipidomic regions of the spectra. Mouse brain imaging data was also collected using the CO_2 laser (C), the OPO laser (D) and the OPA source (E) at different raster sizes.

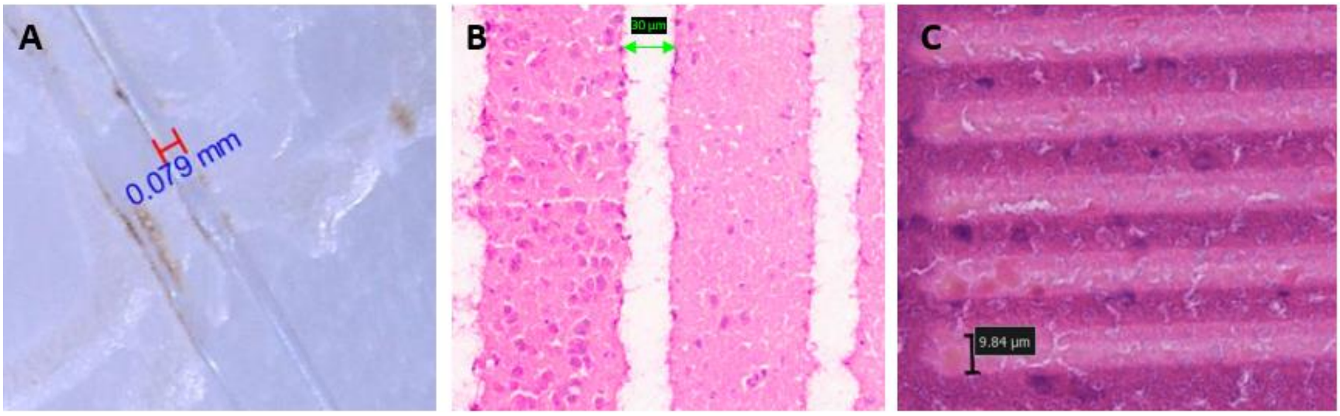

Supplementary Figure S 7: Microscopy image of the CO2 laser (A), the OPO laser (B) and OPA laser (C) tracks after ablation. Around 80 micron spot sizes can be achieved with the CO2 laser at best case scenarios, around 30 micron spots with the OPO laser and sub 10 micron spots can be achived with the picosecond laser.

To challenge the resolution limit of the OPO system, the concept of image oversampling was used. Using an ablation spot size of 30 µm different raster dimensions were tested on consecutive coronal mouse brain sections to determine the best achievable resolution. Oversampling the analytical beam has been proposed and successfully demonstrated using MALDI [3,4]. The images at different pixel sizes (70 µm; 50 µm; 30 µm and 20 µm) are shown in Supplementary Figure S7, revealing the distribution of another nucleobase adenine $[M-H]^-$ at 134.034 m/z. The images show the hippocampal region, and as the pixel sizes decrease the details of the dentate gyrus region become better defined, and the contrast between the polymorph and the granule cell layer increases. At ≤ 30 µm pixel sizes, the fine structures of the stratum radiatum and the stratum lacunosum-moleculare regions in the mouse brain also become observable. Oversampling during the imaging does not affect the irradiation fluence, only the amount of ablated tissue in each spectra, thus no shifts were observed in the ratio of $[M-NH_4]^-$ to $[M-H]^-$ ion intensities under oversampling conditions. The currently achieved resolution limits make the OPO and OPA-coupled LA-REIMS technique competitive (MALDI) or superior (DESI) to other commercially available MSI techniques with regard to resolution. While the higher-resolution images provide superior imaging data, this comes at the price of increased analysis time. The imaging experiment of a coronal mouse brain section at 70 µm took 40 minutes (≈3 cm^2 area) using 10 pixel/second acquisition rate, and a consecutive (thus similar sized) section analysed at 20 µm using the same speed requires more than six hours.

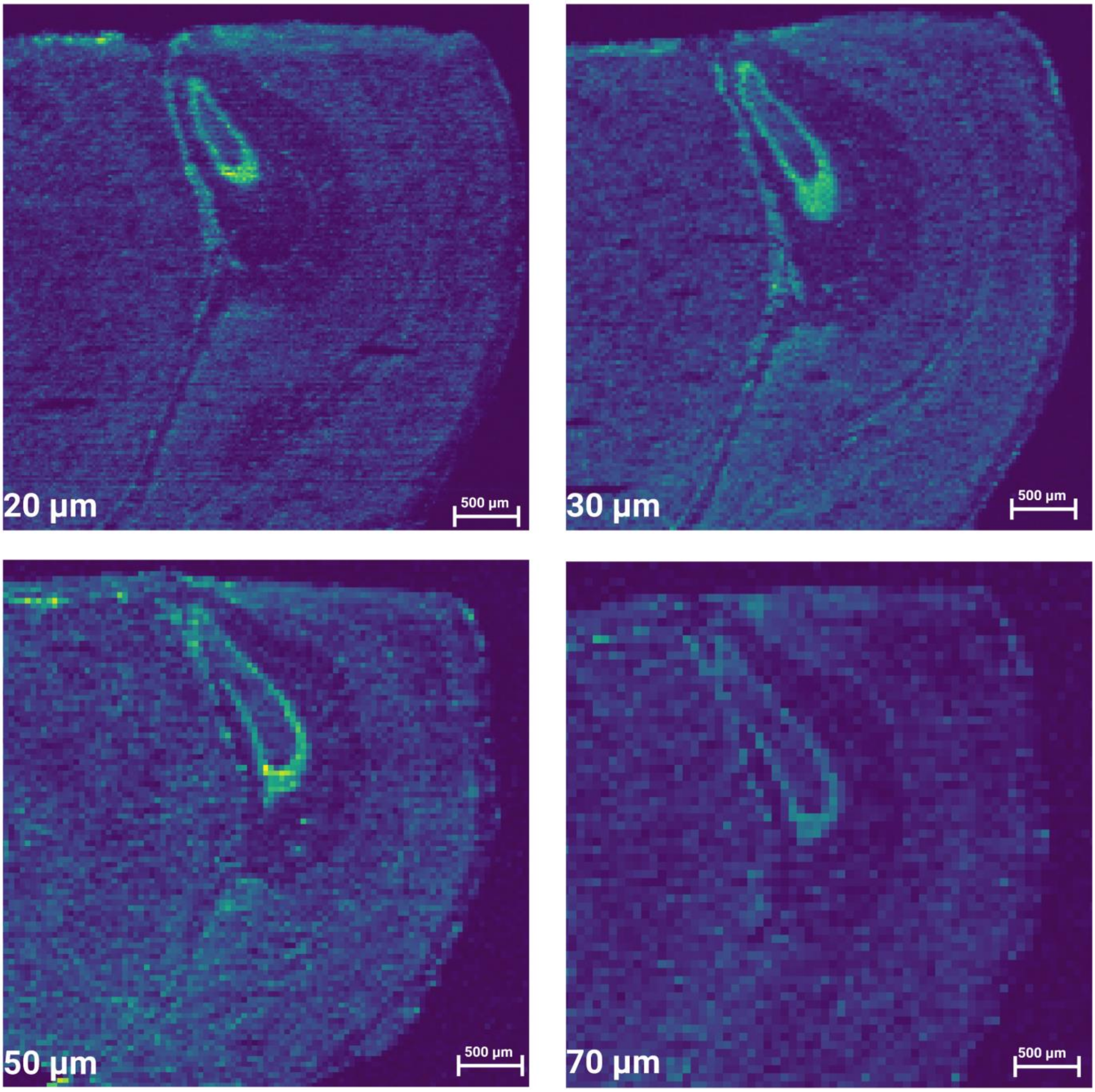

Supplementary Figure S 8: Optimization of laser parameters for imaging experiments using the tuneable OPO laser.

Mouse brain imaging of 134.04 m/z Adenine $[M-H]^-$ was performed using the OPO laser setup with pixel sizes 20 µm (A), 30 µm (B), 50 µm (C) and 70 µm (D) on consecutive tissue sections. The observed features using the molecule Adenine show the detailed features of the hippocampal region of the mouse brain sections.

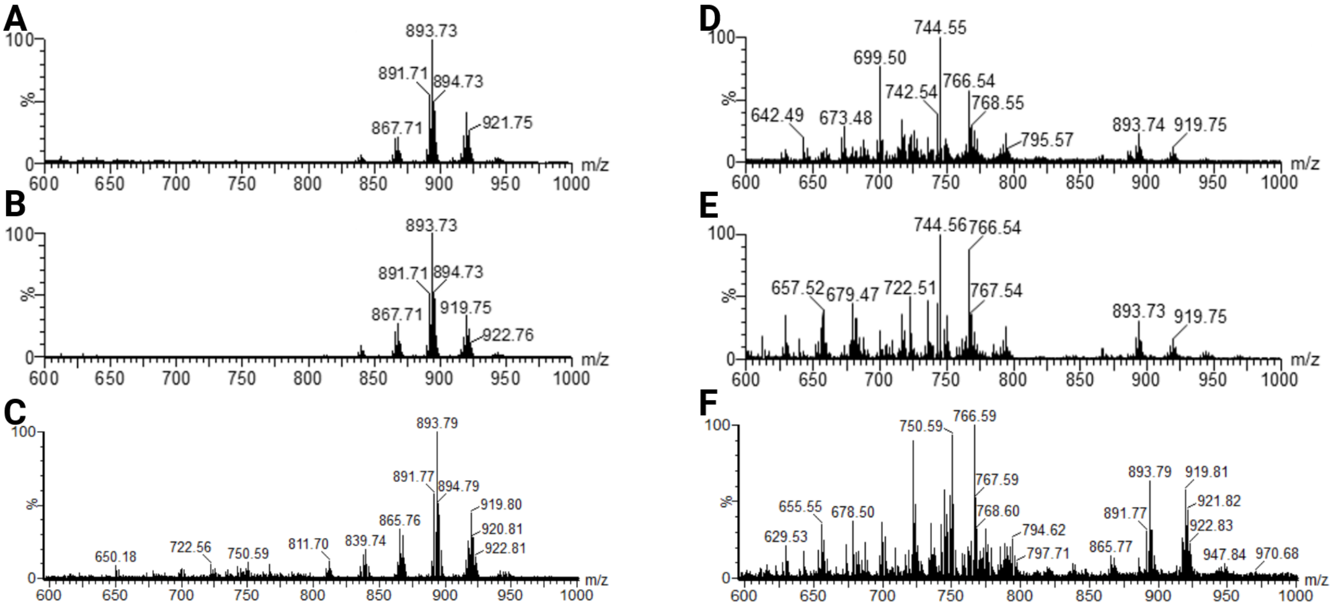

Supplementary Figure S 9: Spectral comparison between healthy breast tissue obtained with the surgical CO2 laser (A), iKnife (B) and OPO laser (C) and cancerous breast tissue sampled with surgical CO2 laser (D), iKnife (E) and OPO laser (F).

MALDI comparison

The performance of the LA-REIMS was compared to MALDI on mouse brain. For LA-REIMS the OPO laser was used for the experiments, operated at 5 J/cm^2, the section was imaged at 40 µm raster size, negative ion mode. For MALDI, 9-aminoacridine (9AA) matrix was chosen as a matrix at negative ion mode. HTX M3+ sprayer was used for the matrix application, with the following experimental parameters: 10 mg/mil 9AA was solved in 80% MeOH – H_2O mix and was loaded into the sprayer. The spray configuration was the following: 70 C spray temperature, 6 passes, 0.08 ml/min flow rate, 1200 mm/min velocity, 3 mm track spacing 10 PSI pressure, 2 l/min flow rate, 2 sec drying time and CC pattern. The results can be observed in Supplementary Figure S10. The MALDI spectra contains 2482 raw features, compared to the REIMS specta s 3171 features, the MALDI contains less features including the prevalent matrix features. The spectral profiles in S10B also confirm this, as the MALDI spectra features less small molecular features compared to the REIMS spectra.

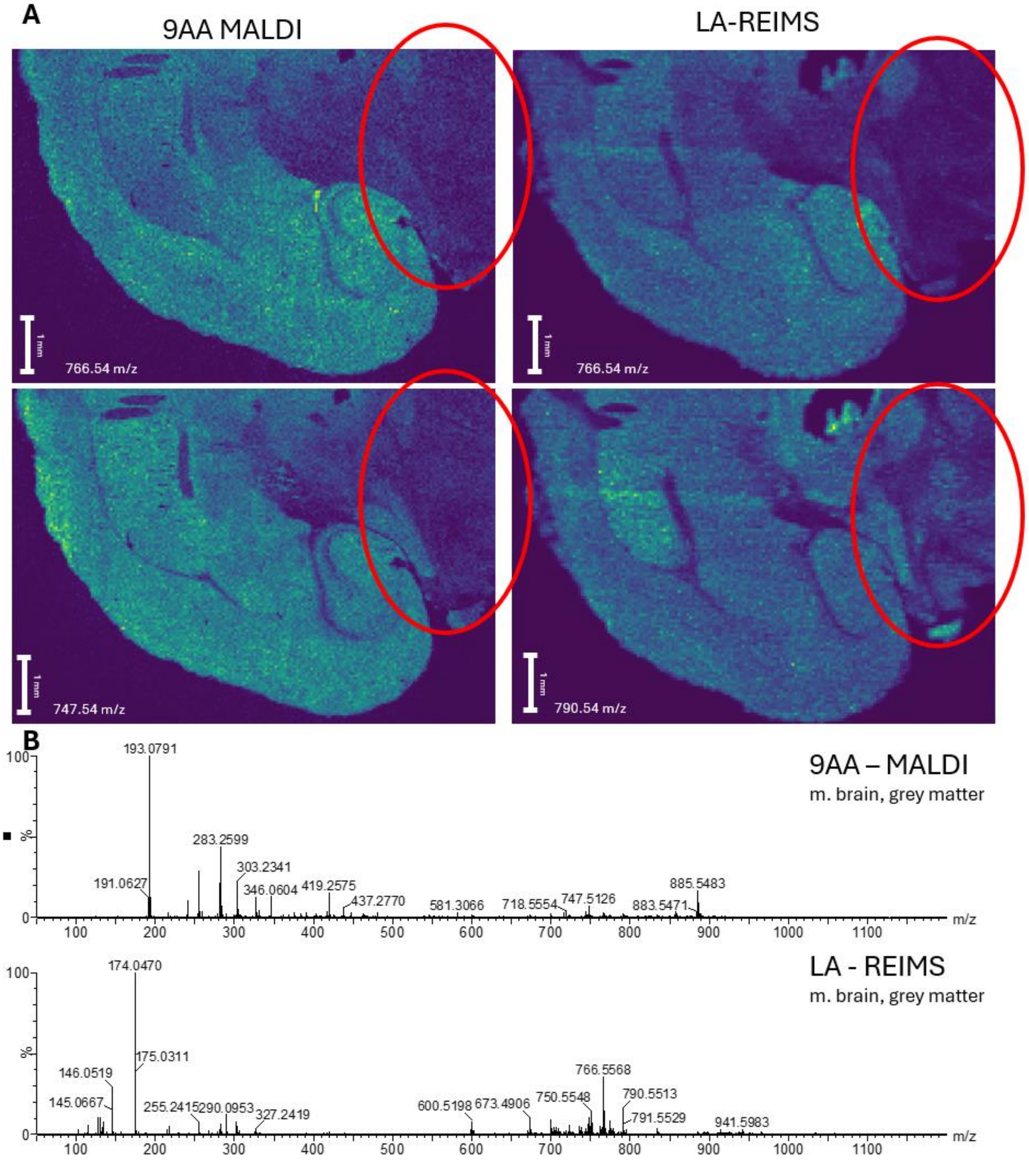

Supplementary Figure S 10: Comparison between negative mode MALDI (using 9AA matrix) and LA-REIMS experiment on mouse brain section. A: imaging comparison between MALDI and LA-REIMS for several phospholipid species, both performed at 40 µm raster sizes. B: single pixel spectral comparison between the MALDI and LA-REIMS spectra. The observed spatial distribution of a given ion are similar between the two modalities, but more low-contrast features can be observed on the REIMS images, hinting at a superior imaging performance when considering similar ionic species.

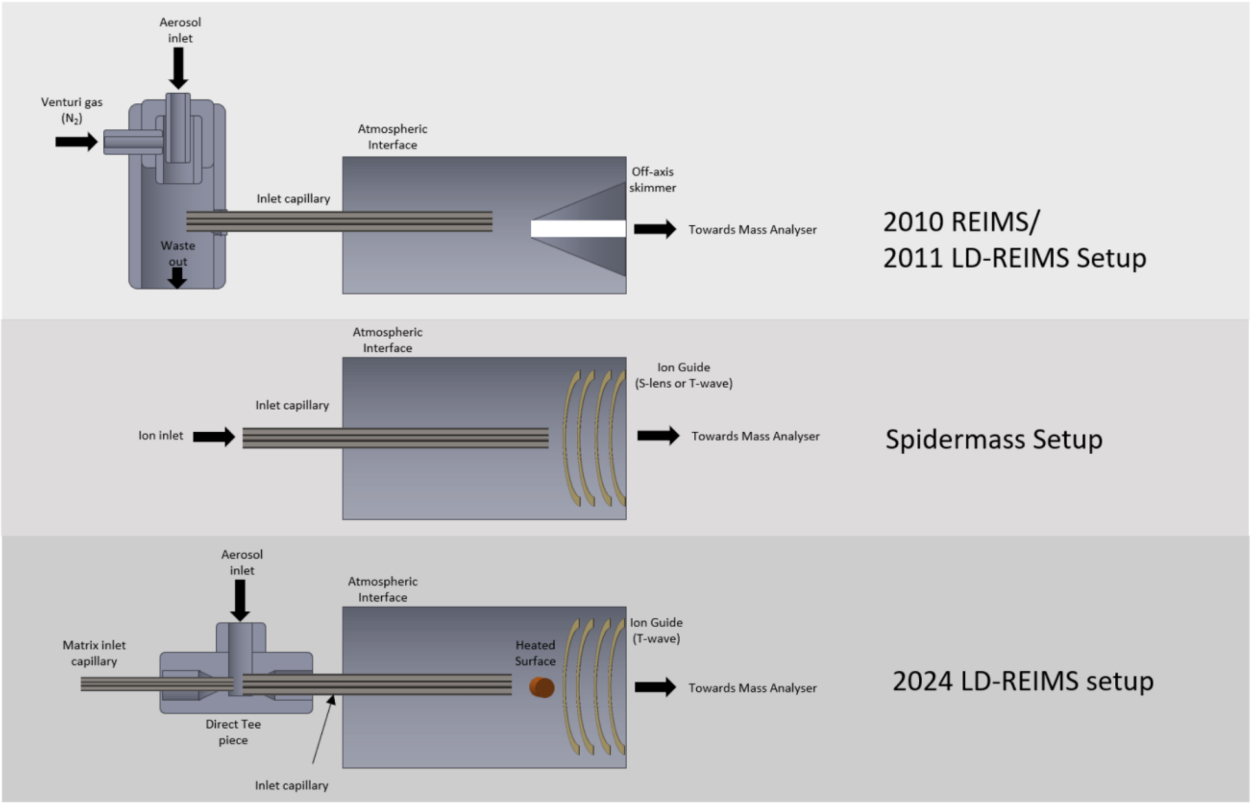

Supplementary Figure S 11: Interface differences between the 2010 REIMS/LD-REIMS interface, the Spidermass interface and current updated LD-REIMS interface. The 2010/2011 LD-REIMS interface uses a Venturi airjet pump to remove the aerosols from the ablation spot coupled to a standard inlet capillary – skimmer interface. The Spidermass setup uses the standard interface geometry of a Thermo Q-Exactive instrument, or a home-built interface on a Synapt G2 instrument equipped with a T-wave ion guide. The bottom figure shows our current LD-REIMS setup in comparison.

Supplementary tables

	CO2	OPO	OPA
Manufacturer	Omniguide	Opotek	Custom design
Model	Intelliguide FEL25SA	HE2731	-
Wavelength	10.6 µm	2.7 – 3.1 µm	2.94 µm
Pulse width	~ 100 microseconds	5-7 nanoseconds	10 picoseconds
Operation mode	Super pulse	Pulsed, 20 Hz	Pulsed, 8.3 MHz

Table S1: Properties and operation mode of the different lasers used for the experiments.

Annotation	Detected ion	Ionic formula	Theoretical m/z	Error ppm	LC-MS/MS	RT	[M+H]+
Aminobutyric acid	$[M-H]^-$	C4H8NO2	102.0555	-5.9	gamma and beta aminobutyric acid confirmed based on retention times and fragment ions with LC-MS/MS		
Asparate	$[M-H_2O-H]^-$	C4H4NO3	114.0191	-5.3			
Malic acid	$[M-H_2O-H]^-$	C4H3O4	115.0031	-5.2			
Taurine	$[M-H]^-$	C2H6NO3S	124.0068	4.8	Taurine DDA2	0.36	
Oxoproline	$[M-H]^-$	C5H6NO3	128.0348	-5.5			could also be Glutamate-H_2O or deaminated glutamine
Asparate	$[M-H]^-$	C4H6NO4	132.0297	-4.5	Yes - confirmation with labelled standard cell lines		
Malic acid	$[M-H]^-$	C4H5O5	133.0137	-3.8			
Adenine	$[M-H]^-$	C5H4N5	134.0467	-5.2			
Glutamine	$[M-H]^-$	C5H9N2O3	145.0619	-6.9	Yes - confirmation with labelled standard cell lines		
Glutamate	$[M-H]^-$	C5H8NO4	146.0459	-6.8	Glutamate DDA3	0.34	
Guanine	$[M-H]^-$	C5H4N5O	150.0416	-4.0			
Aminomuconic acid	$[M-H]^-$	C6H6NO4	156.0297	-2.6			
N-Acetylaspartic acid	$[M-H]^-$	C6H8NO5	174.0403	-2.3			
Ascorbic acid	$[M-H]^-$	C6H7O6	175.0243	5.7			
Glucosamine	$[M+Cl]^-$	C6H13NO5Cl	214.0482	-0.9			
Glucose	$[M+Cl]^-$	C6H12O6Cl	215.0322	-0.5			
Pantothenic Acid	$[M-H]^-$	C9H16NO5	218.1028	-1.4			
Cystathione	$[M-H]^-$	C7H13N2O4S	221.0596	0.9			
Citric acid	$[M+Cl]^-$	C6H8O7Cl	226.9959	-0.9			
Palmitic acid (FA(16:0))	$[M-H]^-$	C16H31O2	255.2324	-2.0			
Inosine	$[M-H]^-$	C10H11N4O5	267.0729	3.4			
Uridine	$[M+Cl]^-$	C9H12N2O6Cl	279.0384	1.8			
Oleic acid ((FA(18:1))	$[M-H]^-$	C18H33O2	281.2481	-0.4			
Malonylcarnitine	$[M+Cl]^-$	C10H17NO6Cl	282.0745	4.3			

Stearic acid (FA(18:0))	$[M-H]^-$	C18H35O2	283.2637	-2.1			
N-Acetylneuraminic acid	$[M-H_2O-H]^-$	C11H16NO8	290.0876	0.0			
Dihydroxystearic acid	$[M-H_2O-H]^-$	C18H33O3	297.243	-0.7			
Adenosine	$[M+Cl]^-$	C10H13N5O4Cl	302.0656	0.3			
arachidonic acid (FA(20:4))	$[M-H]^-$	C20H31O2	303.2324	0.0			
Glutathione	$[M-H]^-$	C10H16N3O6S	306.076	0.0			
eicosenoic acid (FA(20:1))	$[M-H]^-$	C20H37O2	309.2794	1.9			
docosahexaenoic acid (DHA, FA(22:6))	$[M-H]^-$	C22H31O2	327.2324	-0.6			
docosatetraenoic acid (FA(22:4))	$[M-H]^-$	C22H35O2	331.2637	0.6			
LPA(16:0)	$[M-H_2O-H]^-$	C19H36O6P	391.225	-1.0			
MG(20:4)	$[M+Cl]^-$	C23H38O4Cl	413.2459	2.2			
LPA(18:1)	$[M-H_2O-H]^-$	C21H38O6P	417.2406	2.9			
LPA(18:0)	$[M-H_2O-H]^-$	C21H40O6P	419.2563	2.6			
LPE(O-16:1)	$[M-H]^-$	C21H43NO6P	436.2828	0.2	LPE(O-16:1) DDA2	2.15	
LPA(18:0)	$[M-H]^-$	C21H42O7P	437.2668	-1.1			
LPE(16:0)	$[M-H]^-$	C21H43NO7P	452.2777	0.4	LPE(16:0) DDA3	1.9	
LPE(O-18:2)	$[M-H]^-$	C23H45NO6P	462.2985	3.2	LPE(O-18:2) DDA2	2.25	
LPE(O-18:1)	$[M-H]^-$	C23H47NO6P	464.3141	1.9	LPE(O-18:1) DDA1	2.72	
LPE(18:1)	[M-H]-	C23H45NO7P	478.2934	3.3	LPE(18:1) DDA2	2.04	
LPE(18:0)	$[M-H]^-$	C23H47NO7P	480.309	2.5	LPE(18:0) DDA2	2.44	
LPS(18:0)	$[M-H_2O-H]^-$	C24H45NO8P	506.2883	6.9			
Cer(d36:1)	$[M+Cl]^-$	C36H71NO3Cl	600.5122	2.5			
Cer(d38:1)	$[M+Cl]^-$	C38H75NO3Cl	628.5435	2.9			
PA(34:1)	$[M-H]^-$	C37H70O8P	673.4808	3.6			

DG(38:4)	$[M+Cl]^-$	C41H72O5Cl	679.5068	2.5			
Cer(d42:2)	$[M+Cl]^-$	C42H81NO3Cl	682.5905	5.4			
PA(36:2)	$[M-H]^-$	C39H72O8P	699.4965	4.7			
PE(O-34:2)	$[M-H]^-$	C39H75NO7P	700.5281	0.9	PE(O-16:1_18:1) DDA1	7.06	
PA(36:1)	$[M-H]^-$	C39H74O8P	701.5122	21.5	PA(18:1_18:0) DDA2	6.28	
PE(34:1)	$[M-H]^-$	C39H75NO8P	716.523	10.9	PE(16:0_18:1) DDA3	6.68	
PE(34:0)	$[M-H]^-$	C39H77NO8P	718.5387	2.2			
PE(O-36:5)	$[M-H]^-$	C41H73NO7P	722.5125	1.9			
PE(O-36:3)	$[M-H]^-$	C41H77NO7P	726.5438	5.9	PE(O-18:2_18:1) DDA1	7.14	
PE(O-36:2)	$[M-H]^-$	C41H79NO7P	728.5594	3.3			
PE(36:4)	$[M-H]^-$	C41H73NO8P	738.5074	-0.1	PE(16:0_20:4) DDA3	6.16	
PE(36:2)	$[M-H]^-$	C41H77NO8P	742.5387	7.3	PE(18:1/18:1) DDA1	6.76	
PE(36:1)	$[M-H]^-$	C41H79NO8P	744.5543	4.7	PE(18:1_18:0) DDA1	7.31	
PE(O-38:7)	$[M-H]^-$	C43H73NO7P	746.5125	6.7	PE(O-16:1_22:6) DDA1	6.33	
PE(O-38:6)	$[M-H]^-$	C43H75NO7P	748.5281	0.0	PE(O-18:2_20:4) DDA1	6.58	
PE(O-38:5)	$[M-H]^-$	C43H77NO7P	750.5438	3.3	PE(O-18:1_20:4) DDA1	7.19	
PE(O-38:4)	$[M-H]^-$	C43H79NO7P	752.5594	-2.4	PE(O-18:1_20:3) DDA3	7.36	
PE(O-38:3)	$[M-H]^-$	C43H81NO7P	754.575	5.6	PE(O-18:1_20:2) DDA4	7.67	
PE(O-38:2)	$[M-H]^-$	C43H83NO7P	756.5907	4.2	PE(O-18:1_20:1) and PE(O-18:0-20:2)	8.18	
PS(34:0)	$[M-H]^-$	C40H77NO10P	762.5286	-6.8	PS(16:0_18:0) DDA2	5.97	
PE(38:5)	$[M-H]^-$	C43H75NO8P	764.523	16.2	PE(18:1_20:4) DDA1	6.29	
PE(38:4)	$[M-H]^-$	C43H77NO8P	766.5387	4.8	PE(18:0_20:4) DDA2	6.88	
PE(38:2)	$[M-H]^-$	C43H81NO8P	770.57	3.6			
PG(36:2)	$[M-H]^-$	C42H78O10P	773.5333	2.8			
PE(O-40:7)	$[M-H]^-$	C45H77NO7P	774.5438	3.9	PE(O-18:1_22:6) DDA1	6.97	
PG(36:1)	$[M-H]^-$	C42H80O10P	775.5489	1.8			
PE(O-40:6)	$[M-H]^-$	C45H79NO7P	776.5594	0.3	PE(O-18:2_22:4) DDA2	7.05	
PG(36:0)	$[M-H]^-$	C42H82O10P	777.5646	3.7			
PE(O-40:5)	$[M-H]^-$	C45H81NO7P	778.5751	-0.4	PE(O-18:1_22:4) DDA1	7.57	

PE(O-40:3)	$[M-H]^-$	C45H85NO7P	782.6064	-11.5	PE(O-18:2_22:1) DDA3	8.22	
PS(36:2)	$[M-H]^-$	C42H77NO10P	786.5285	-6.5	PS(18:1/18:1) DDA3	5.39	
PS(36:1)	$[M-H]^-$	C42H79NO10P	788.5442	-11.3	PS(18:1_18:0) DDA3	5.95	
PE(40:6)	$[M-H]^-$	C45H77NO8P	790.5387	9.2	PE(18:0_22:6) DDA1	6.63	
PE(40:4)	$[M-H]^-$	C45H81NO8P	794.57	1.5	PE(18:0_22:4) DDA2	7.26	
PC(O-36:1)	$[M+Cl]^-$	C44H88NO7PCl	808.5987	6.8	PC(O-20:1_16:0)	7.47	774.6377
HexCer(d40:1)	$[M+Cl]^-$	C46H89NO8Cl	818.6277	3.9			784.6666
PS(40:6)	$[M-H]^-$	C46H77NO10P	834.5285	-7.4	PS(18:0_22:6) DDA3	5.26	
PC(O-38:2)	$[M+Cl]^-$	C46H90NO7PCl	834.6144	18.2	PC(O-20:1_18:1)	8.02	800.6533
PI(34:1)	$[M-H]^-$	C43H80O13P	835.5337	-11.6	PI(16:1_18:0) and PI(16:0_18:1)	5.49	
PC(O-38:1)	$[M+Cl]^-$	C46H92NO7PCl	836.63	4.1			802.669
HexCer(d42:2)	$[M+Cl]^-$	C48H91NO8Cl	844.6433	7.6	HexCer(d18:1/24:1) DDA1 pos	7.94	810.6823
HexCer(d42:1)	$[M+Cl]^-$	C48H93NO8Cl	846.659	-1.4	HexCer(d18:1/24:0) DDA1 pos	8.54	812.6979
SM(d42:2)	$[M+Cl]^-$	C47H93N2O6PCl	847.646	16.4	SM(d18:1_24:1) DDA1 pos	7.71	813.6849
SM(d42:1)	$[M+Cl]^-$	C47H95N2O6PCl	849.6616	-7.8			815.7006
PE(44:3)	$[M-H]^-$	C49H91NO8P	852.6482	6.9			
PC(O-40:1)	$[M+Cl]^-$	C48H96NO7PCl	864.6613	1.7			830.7002
PG and/or BMP(44:12)	$[M-H]^-$	C50H74O10P	865.502	1.3	BMP(22:6/22:6) DDA5	4.01	867.5176
PC(O-40:0)	$[M+Cl]^-$	C48H98NO7PCl	866.677	-8.3			832.7159
PS(44:0)	$[M-H]^-$	C48H93NO10P	874.6538	-6.3			
PI(38:4)	$[M-H]^-$	C47H82O13P	885.5493	15.7	PI(18:0_20:4) DDA1	5.43	

Table S2.: List of annotated peaks observed during the imaging experiments in mouse brain sections.

	**170 µs**	**190 µs**	**210 µs**	**230 µs**
ZnSe12	2 mW on 46 µm spot	1.9 mW on 52µm spot	1.3 mW on 50µm spot	0.45 mW on 50µm spot
ZnSe25	2,5 mW on 70 µm spot	2.1 mW on 70 µm spot	1.5 mW on 70 µm spot	0.45 mW on 70 µm spot
CaF2	2,4 mW on 70 µm spot	2.0 mW on 70 µm spot	1 mW on 70 µm spot	0.45 mW on 70 µm spot
2xCaF2	1,7 mW on 65 µm spot	1.6 mW on 65 µm spot	1 mW on 65 µm spot	0.45 mW on 65 µm spot

Table S3.: List of lenses and measured power values used for the fluence experiments.

References

(1) Jugroot, M.; Groth, C. P. T.; Thomson, B. A.; Baranov, V.; Collings, B. A. Numerical Investigation of Interface Region Flows in Mass Spectrometers: Neutral Gas Transport. J Phys D Appl Phys 2004, 37 (8), 1289–1300. https://doi.org/10.1088/0022-3727/37/8/019.

(2) Schäfer, K.-C.; Dénes, J.; Albrecht, K.; Szaniszló, T.; Balog, J.; Skoumal, R.; Katona, M.; Tóth, M.; Balogh, L.; Takáts, Z. In Vivo, In Situ Tissue Analysis Using Rapid Evaporative Ionization Mass Spectrometry. Angewandte Chemie International Edition 2009, 48 (44), 8240–8242. https://doi.org/10.1002/anie.200902546.

(3) Maimó-Barceló, A.; Garate, J.; Bestard-Escalas, J.; Fernández, R.; Berthold, L.; Lopez, D. H.; Fernández, J. A.; Barceló-Coblijn, G. Confirmation of Sub-Cellular Resolution Using Oversampling Imaging Mass Spectrometry. Anal Bioanal Chem 2019, 411 (30), 7935–7941. https://doi.org/10.1007/s00216-019-02212-3.

(4) Jurchen, J. C.; Rubakhin, S. S.; Sweedler, J. V. MALDI-MS Imaging of Features Smaller than the Size of the Laser Beam. J Am Soc Mass Spectrom 2005, 16 (10), 1654–1659. https://doi.org/10.1016/j.jasms.2005.06.006.
